# Revealing Yukon’s hidden treasure: an atomic-scale investigation of Carlin-type gold mineralization in the Nadaleen Trend, Canada

**DOI:** 10.1007/s00126-024-01325-9

**Published:** 2024-10-30

**Authors:** Phillip Gopon, Patrick Sack, Nicolas Pinet, James O. Douglas, Benjamin M. Jenkins, Brooke Johnson, Ella Penny, Michael P. Moody, Laurence Robb

**Affiliations:** 1 Department of Applied Geosciences and Geophysics, Montanuniversität Leoben, 8700 Leoben, AT Austria; 2 Department of Materials, University of Oxford, Oxford, OX1 3PH UK; 3 Department of Earth Sciences, University of Oxford, Oxford, OX1 3AN UK; 4 Yukon Geological Survey, Whitehorse, Yukon, CA Y1A 2C6 UK; 5 Natural Resources Canada, Geological Survey of Canada, Quebec, CA G1K9A9 Canada; 6 Department of Materials, Imperial College London, London, SW7 2AZ6 UK; 7 University Rouen Normandie, CNRS, INSA Rouen Normandie, Groupe de Physique des Matériaux UMR 6634, F-76000 Rouen, France; 8 Department of Geology, University of Liege, 4000 Liege, BE Belgium

## Abstract

The invisible-gold deposits known as Carlin-type are becoming more important as easier to find deposits are progressively depleted. The combination of the invisible nature of the Au in these deposits, as well as the limited surface indicators of these deposits, makes exploration to find new Carlin-type deposits extremely difficult. Comprehensive mineralization models are essential to find new Carlin-type deposits in similar geologic settings. The Nadaleen Trend of Yukon, Canada, is one such district where an improved understanding of this deposit type has led to new discoveries. Previous studies compared and contrasted the tectonic setting, host rock depositional setting, structural preparation, and mineralization style of the Nadaleen Trend with those in Carlin-type localities, Nevada. However, the comparisons at an atomic scale, between Carlin-type Au deposits in the Nadaleen Trend and those in Nevada, has yet to be investigated. This study fills this knowledge gap by combining high resolution microanalytical techniques with atom probe tomography to examine the distribution of Au and other trace elements in the Nadaleen Trend, compare them to a representative Carlin-type deposit in Nevada (Turquoise Ridge), and determine how widespread the mineralization model is. Our findings show that in the Nadaleen Trend, as in Nevada, Au is generally directly linked with As at the macro to atomic scale, and is incorporated into As/Au rich overgrowths on sedimentary/diagenetic pyrite. Gold-rich pyrite rims in the Nadaleen Trend are generally smaller than those found in Nevada (0.5–2 µm vs > 10 µm), although the ore grades appear comparable. We find that the Au in the pyrite of the Nadaleen Trend is homogenously distributed (i.e. lattice bound) at the atomic scale, but that there is a notable enrichment of As surrounding individual Au atoms. These findings are in agreement with those from previous work on a representative deposit in Nevada, and support the assertation that As is the key ingredient in facilitating the incorporation of Au into the pyrite lattice. Arsenic as an essential component in the trapping mechanisms of Au in CTG deposits, is something that has been as to yet underappreciated in the current models of CTG deposit formation.

**Supplementary Information:**

The online version contains supplementary material available at 10.1007/s00126-024-01325-9.

## Introduction

The Nadaleen Trend (Yukon, Canada) is located ~ 3,000 km N-NW of the Carlin Trend (Nevada, USA) for which Carlin-type gold (CTG) deposits are named. The discovery of CTG mineralization in the Nadaleen Trend is notable because CTG deposits are not well known outside of Nevada (Xie et al. 2018), but also because of the lithological, geochemical, and tectonic similarities to those in the Nevadan CTG deposits (Pinet and Sack 2019; Pinet et al. 2022a, b and c, 2023; Steiner and Hickey 2022, 2023). The Yukon and Nevadan CTG deposits are hosted in similar carbonate-rich lithologies, are both associated with regional fault structures, exhibit similar alteration and pathfinder elements, and Au in both regions is associated with pyrite (Cline et al. 2005; Muntean et al. 2011; Pinet et al. 2022c). However, mineralization in the Nevadan deposits is associated with magmatism related to a shift from compression to extension (Muntean et al. 2011), while the mineralization in Yukon has not been linked to volumetrically significant magmatism and formed under a transpressional tectonic regime (Pinet et al. 2022b). Despite these similarities and differences between Yukon and Nevadan CTG deposits at the regional to micrometer scale, the question as to the similarities and differences at the nanoscale and the atomic scale (i.e. how Au specifically occurs and is incorporated into pyrite) remains unanswered.

Gopon et al. (2019a) showed, using atom probe tomography (APT), that the Turquoise Ridge/Getchell deposit in Nevada (taken to be a representative CTG deposit; Cline 2001) is characterized by homogenously distributed, structurally bound Au, that is intimately associated with As at the atomic scale. The replacement of sulfur with As in the pyrite structure leads to lattice defects and partial charge imbalances such that, when ~ 200 As ions replace sulfur, this creates a local 1^−^ charge that can be neutralized by Au^1+^ (Reich et al. 2005; Deditius et al. 2014; Gopon et al. 2019a; Kusebauch et al. 2019; Pokrovski et al. 2019). The questions as to how widespread or important this incorporation process is, and the importance of As in the formation of CTG, are still debated. An alternative explanation for Au incorporation in CTG is that nanoparticles of Au are trapped in pyrite and/or arsenopyrite during rapid growth with little or no link to As required (Palenik et al. 2004; Barker et al. 2009; Fougerouse et al. 2016; Wu et al. 2021).

This paper sets out to compare the Nadaleen Trend CTG deposits and those in Nevada (using the Turquoise Ridge/Getchell deposit as a representative example) from the micrometer to atomic scale. Of particular interest is the role of structurally bound/As-linked Au. By investigating geographically distant, but geologically similar deposits, we find that the Au-As relationship is integral to the formation of CTG deposits.

## Geologic framework

**Fig. 1:**
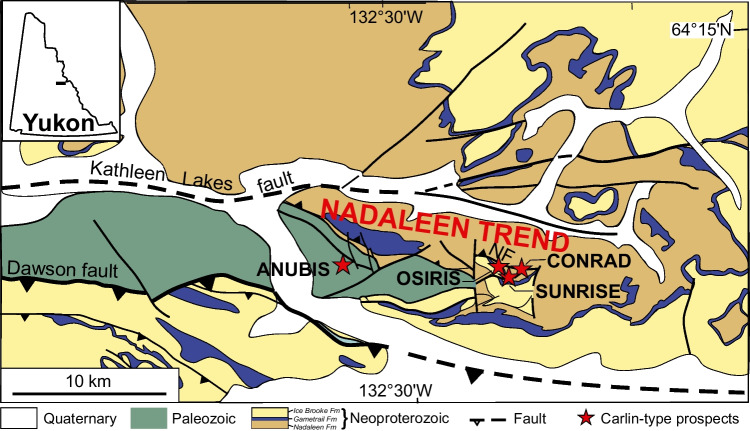
Simplified geologic map of the Nadaleen Trend and the location of the main deposits (from Pinet et al. 2022a, b and c; see Moynihan, 2016 for detailed geologic map). NF = Nadaleen Fault

**Fig. 2:**
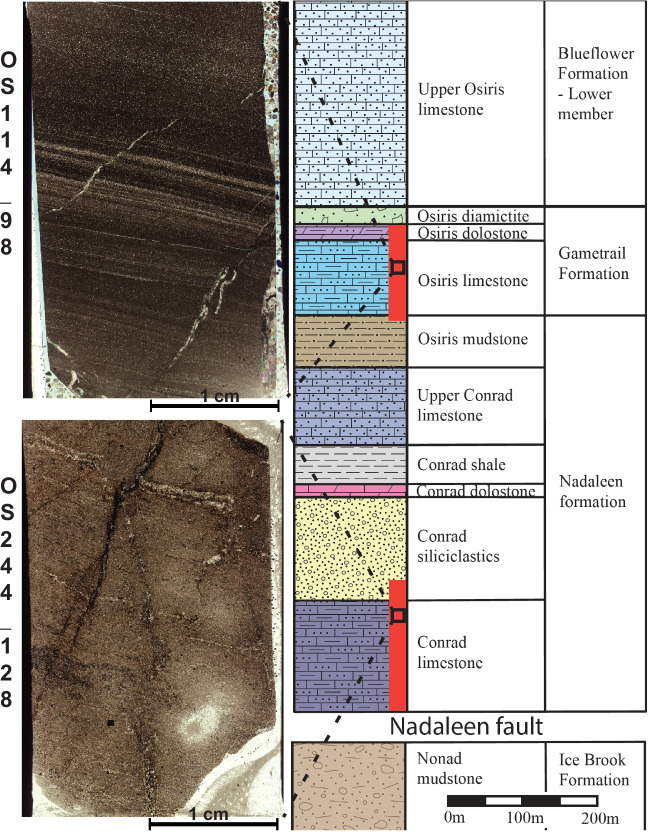
Location of samples used in the APT study within a simplified stratigraphic column (modified from Steiner et al. 2017), as well as thin section image of the samples OS114_98 and OS244_128. Red lines in the stratigraphic column indicate mineralization

The tectonic and sedimentary frameworks of the Nadaleen trend is described in detail by Arehart et al. (2013), Moynihan et al. (2019), Pinet et al. (2022b and 2023) and Steiner et al. (2017) among others. In summary, the Nadaleen Trend is located in the foreland belt of the North American Cordillera, which consists of sedimentary rocks deposited on the Laurentian (North American) passive margin at the edge of the rifted Rodinian supercontinent. The Yukon CTG deposits are located within the WNW-ESE trending Nadaleen trend, a strip of Neoproterozoic to Permian sedimentary rocks bound by the regional deep-seated Dawson fault to the south, a long-lived structural feature that controlled the transition zone between the Ogilvie Platform and the Selwyn Basin, and by the Kathleen Lakes fault to the north (Pinet et al. 2022c). The Nadaleen Trend consists of three CTG deposits hosted in three distinct formations: Anubis, Conrad, and Osiris-Ibis-Sunrise (Figs. 1, and 2).

Resource calculations for the Conrad and Osiris-Ibis-Sunrise deposits yielded a total indicated and inferred resource of ca. 14.9 Mt at an average grade of 3.71 g/t Au, for a total of 1.78 Moz (ATAC-Resources 2022). The Au mineralization is roughly concordant with the bedding and confined to steeply dipping units involved in tight anticlines and overlain by impermeable rock packages or faults. In Neoproterozoic hosted deposits (including the Conrad and Osiris-Ibis-Sunrise deposits), mineralized units correspond mainly to thin bedded limestones and debris flow deposits, with few mineralized intervals in carbonaceous siltstones. The ore-forming sequence includes an early stage characterized by decarbonatization, an intermediate stage characterized by Au-bearing pyrite and silicification, and a late-stage mineral assemblage including blocky calcite, realgar, orpiment and fluorite. Timing of the Au mineralization is constrained at ca. 74 Ma by U–Pb dating of calcite veins that cross cut the mineralized zones, and give a minimum age of formation of 74–71 Ma (Pinet et al. 2022b). Furthermore, two mafic dikes at the Conrad deposit have been dated by U–Pb in zircon to 74 Ma (Tucker et al. 2018). These dikes are locally altered by ore fluids, and therefore represent a maximum age of mineralization. However, this 74 Ma age has not yet been temporally linked with any larger scale intrusions in the area. At the regional scale, the most voluminous intrusive suites in the Yukon, east of the Tintina fault, are dated at 98–90 Ma and are associated with intrusion related gold and tungsten skarn deposits (Hart et al. 2004). The younger (ca. 74 Ma) Yukon CTG deposits are possibly associated with hydrothermal fluids linked to one or several buried intrusion(s) contemporaneous with the dated mafic dikes, or associated with a different source of hydrothermal fluids (Pinet et al. 2022a).

In this study, we use a previously well characterized sample from the Turquoise Ridge underground mine to represent an ‘average’ CTG deposit in Nevada (Cline and Hofstra 2000; Cline 2001; Gopon et al. 2019a). In Nevada, CTG deposits occur along a series of three N-NW trending (Battle Mountain-Eureka, Carlin, and Alligator Ridge), and two N-NE trending (Getchell and Jerritt Canyon) mining trends. Mineralization is generally found along large high-angle regional faults, especially below the shallowly dipping Roberts Mountain Thrust, but can also occur as stratabound mineralization. Mineralization is preferentially hosted in Devonian to Mississippian silty carbonates and clastic sedimentary units, although there are examples of mineralization hosted in dikes or along the margin of metamorphic contact aureoles (Arehart et al. 2013). Proven and probable reserves at the Turquoise Ridge/Getchell deposit have an average ore grade of 6.71 g/ton that represents a midpoint in the range of grades across CTG deposits (Cline et al. 2005; Berger et al. 2014). Mineralization at Turquoise Ridge/Getchell occurs along the regional scale Getchell fault with mineralization in the Turquoise Ridge underground section being additionally locally controlled by a dacite dyke. Host rocks are silty carbonates from the Cambrian Preble and Ordovician Comus Formation. Turquoise Ridge/Getchell has, in the past, been used as an ‘average’ or representative CTG deposit in terms of its mineralization style (Cline and Hofstra 2000; Barker et al. 2009; Gopon et al. 2019a), but we acknowledge the issues and limitations that arise from this assumption.

The comparison of Yukon CTG with those of Nevada brings to light many similarities in the regional settings, structural ground preparations, host rock lithologies, alteration styles, mineralizing fluid temperatures, pathfinder elements and Ag/Au ratios (see the Table 1 of Pinet et al. 2023 for details and references). The main differences are the spatial and temporal relationships with magmatism. The Nevadan CTG deposits have been temporally and isotopically linked to extensional magmatism (Muntean et al. 2011; Holley et al. 2022), although this remains debated with a regional/orogenic fluid source being suggested as an alternative explanation (Large et al. 2011). The spatial and temporal relationships between CTG mineralization and magmatism are far less clear in Yukon.

## Methods

### Sample selection / petrography

A total of 20 samples from 5 boreholes, were investigated from the Osiris-Ibis-Sunrise and Conrad deposits (Fig. 1, Fig. 2). These samples were collected for a previous study, and the ore stage sulfides where geochemically and microscopically investigated by Sack et al. (2019). A high-resolution petrographic investigation of a subset of four of these samples was carried out in this study, which included reflected light microscopy and scanning electron microscopy using a ‘FEI- Quanta 650’ field emission gun (FEG) sourced scanning electron microscope (SEM). These 4 representative samples were also re-investigated by transmitted/reflected light petrography with a particular emphasis on the pre-ore pyrite and the sedimentary textures. Optical petrography was carried out using a Zeiss Axio petrographic microscope fitted with a Zeiss Axiocam, and images were captured with Zeiss Axio software. The high-resolution petrographic investigation was combined with the initial work of Sack et al. (2019), to identify a representative sample from each of the two deposits for further characterization at the micrometer scale (i.e. high resolution EPMA mapping).

### High-resolution EPMA

**Fig. 3:**
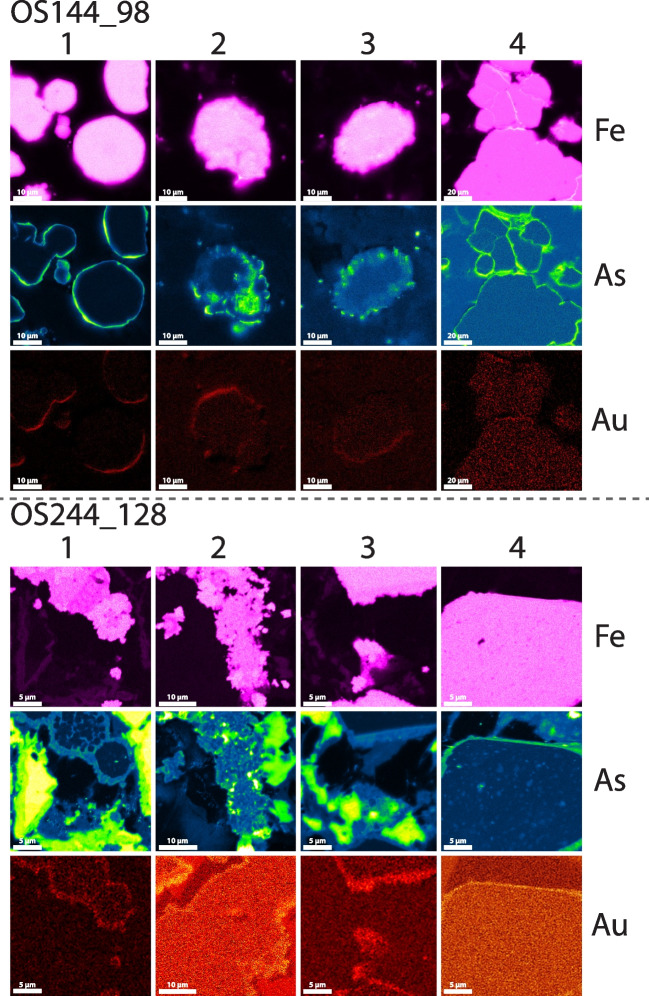
High-resolution EPMA maps of gold containing pyrites from the Conrad (top) and Osiris-Ibis-Sunrise (bottom) deposits

Polished thin sections from the two selected samples (OS114_98: Conrad, borehole 114, 97 m; OS244_128: Osiris-Ibis-Sunrise, borehole 244, 128 m; ESM Fig. 1) were further investigated with the aid of a ‘CAMECA- SX-5’ FEG sourced electron probe microanalyzer (EPMA) located in the Dept. of Earth Sciences at the University of Oxford. Overview (faster/lower-resolution) EPMA maps of pyrite were conducted using high beam current (200nA), high accelerating potential (25keV), and a shorter pixel dwell time of 0.1s, to increase the signal and allow fast mapping of a large number of pyrite grains (~ 1 h per map). Using these initial maps, a small subset of those regions, where re-mapped at high resolution, to visualize the sub-micrometer scale distribution of Au, As, S, and Fe within the pyrite. These second maps were acquired at 10 keV, 50 nA, and a pixel dwell time of 0.3s. Due to the low abundance of Au, all five wavelength dispersive spectrometers (WDS) were tuned to Au peaks (Lα at 25 keV and Mα at 10 keV) and the signals summed following the analytical protocols set out in Gopon et al. (2019a). All other elements were simultaneously measured on an energy dispersive spectrometer (EDS). Background levels are difficult to quantify in EPMA maps, but because quantitative analysis had been done by Sack et al. (2019), we can estimate that based on the features that were visible in the maps, the detection limits are approximately 150 ppm Au for the fast (low resolution) maps and around 100 ppm Au for the slow (high resolution) maps. EPMA maps for samples OS114_98 and OS244_128 are presented in Fig. 3.

The small size and tendency of pyrites to stand above the gangue minerals during polishing led to increased signal intensity for the rims due to ‘edge effects’ (Jercinovic et al. 2012). ESM Fig. 5 shows this effect visually, in that the ‘Au rich’ region is located on opposite sides of the grain depending on which WDS spectrometers signal is shown. The detector facing the edge obtains more signal and the opposite detector has lower signal due to the signal being ‘shadowed’ by the grain. As we used 5 WDS spectrometers, this effect can be decreased when all 5 WDS signals are summed, but never removed due to the different signal strength of the individual spectrometers (i.e. large vs. small crystal spectrometers, older vs. newer crystals, spectrometer that is slightly out of alignment, etc.).

To decrease sample contamination during these long analyses, which has been shown to have a negative effect on the signal especially at low voltages (Gopon et al. 2013, 2015), a liquid nitrogen cooled cold finger was used during all analyses. The analytical volume of the 10 keV EPMA beam was estimated at 300nm using a combination of modeling and indirect measurement of the electron beam diameter (using the ‘CASINO’ software, Drouin et al. 2007; and the ‘Electron beam measure tool’, Gopon and Sobol 2014).

### Atom probe tomography

**Fig. 4:**
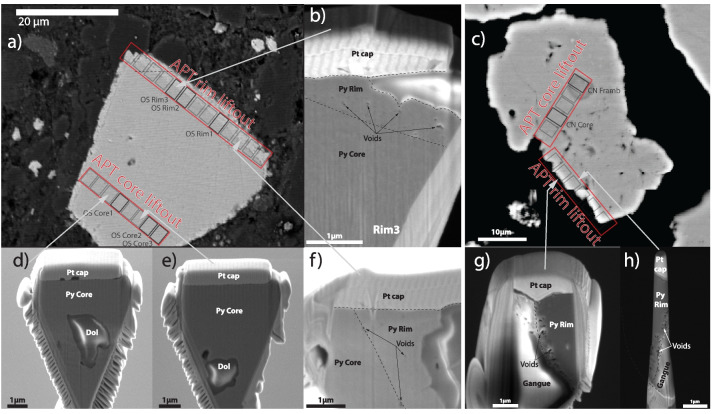
Backscattered electron images showing the location of APT liftout bars from the Osiris-Ibis-Sunrise pyrite (OS244_128; a) and the Conrad pyrite (OS114_98; c). Insets b, d, and e show notable features in the Osiris-Ibis-Sunrise pyrite that were visible during the FIB-SEM sample preparation (sample shown in profile view) including dolomite inclusions as well as voids in the pyrite rims. Insets g and h show notable features in the Conrad pyrite that were visible during the FIB-SEM sample preparation (sample shown in profile view); note the increased number of voids in the rim compared to the Osiris-Ibis-Sunrise pyrite rim. No successful APT datasets were acquired from the void-heavy Conrad rim

One representative Au containing pyrite grain was selected from each of the two samples, for atomic scale characterization. Two APT liftout bars were taken from each pyrite grain, one from the barren core and one from the Au-As rich rim from Conrad (OS114_98) and Osiris-Ibis-Sunrise (OS244_128), using a Zeiss Crossbeam focused ion beam (FIB) SEM at the Dept. of Materials at the University of Oxford. The general FIB-SEM protocols for APT in Thompson et al. (2007), and the geology specific ones in Gopon et al. (2019b), were used during the sample preparation. From each of the four liftout bars, on average 10 APT needle shaped specimens were made that were individually analyzed using APT. Needle shaped specimen geometry was targeted to be standardized at a shank angle of ~ 5° (Gopon et al. 2022), in an attempt to ensure the local environment of each tip was as similar as possible during the APT experiments. Location of liftout bars as well as approximate location of the successful APT experiments are noted in Fig. 4.

APT analyses were conducted on a ‘CAMECA- LEAP 5000XR’ in the Dept. of Materials (University of Oxford) in laser pulsing mode with a 355-nm laser. APT analysis conditions were taken and adapted from previous work by the authors on pyrite that were shown to maximize sample yield, as well as data quality (Gopon et al. 2019a, 2022; Xie et al. 2024). During the run the specimen temperature was kept at 50 K, the laser pulse rate at 125-kHz, laser energy at 80-pJ, and the detection rate at 0.005 average ions per pulse.

Reconstruction of the APT datasets was conducted using the tip-profile mode in the IVAS software (v3.8.8). Identification of the mass spectrum peaks (‘ranging the data’) was also done using the IVAS software (Larson et al. 1999), with the validity of the peak identification verified by a combination of the ‘weights’ script (Haley 2018a), as well as the MATLAB script Atom ProbeLab (London 2020). The AtomProbeLab Matlab ‘bulk decomp’ script (London 2019) was used to calculate concentrations due to its improved complex peak overlap solver (compared to the IVAS software; see Gopon et al. [2022] for further details). For more accurate comparison, the existing APT data from Nevada (Gopon et al. 2019a) was reprocessed with the ‘AtomProbeLab’ software using same range IDs and widths as for the Yukon data. Reported errors in the APT data are based on the level of uncertainty of the overlap correction, which has been shown to be the largest quantifiable source of error in APT compositional data analysis (London 2019). We define our lower limits of detection by lower error limits that are below 5 ppm.

All 3-D atomic scale spatial analysis reported here were done using the 3-Depict software (v0.0.21) by Haley (2018b). Spatial correlations plots are reported as the number of target ions that have a relative concentration of the specified ion within a given volume surrounding each target ion (i.e. the number of Au atoms with a given concentration of As in the 3 nm surrounding volume). These are plotted as normalized to 1, with the lines smoothed, and each point on the line corresponding to the relative number of target ions that contain that concentration of the specified element (Gopon et al. 2019a; Bertrandsson Erlandsson et al. 2023).

All data is reported both as that which was experimentally observed (titled: ‘real’) and with the ion labels randomly reassigned (titled ‘random’). This randomization shows the expected distribution of elements if there was no correlation between the target and specified elements (i.e. what the randomly-distributed matrix would look like). Deviations to the left or right of the randomized curve show the variance from a totally random distribution of the ions. Shifts to the left of the ‘real’ compared to the ‘random’ data therefore represents an anticorrelation between these elements, and shifts to the right of the ‘real’ compared to the ‘random’ data represents a positive correlation between these elements. No shift between the ‘real’ and ‘randomized’ data curves means the elements in the real dataset are randomly distributed and are not spatially correlated or anti-correlated.

## Results

### Petrography / EPMA

Of the samples re-investigated (OS244_128, OS144-98, OS01-80, OS11_50) for their sedimentary structures and occurrence of sedimentary/diagenetic pyrites, the following petrographic relationships were noted:

Sample OS244_128 contains crinkled, anastomosing organic-rich laminae which may represent benthic microbial mats (ESM Fig. 6, inset a). Sample OS114_98 is a finely laminated siltstone that appears to have undergone intense silicification and features veins and spaces filled with quartz and/or chalcedony. Pyrite is abundant in the sample including in the veins, where it appears to postdate the vein lining quartz. Many of the larger pyrite grains feature a spongy and degraded texture, and the Au-bearing rims also show the same spongy texture. This sample does not host any observable realgar, though some veins are associated with poorly crystalline Fe-oxides. On the basis of these petrographic relationships, we identified several generations of pyrite within the examined samples; i) framboidal pyrite, ii) diagenetic pyrite, and iii) late Au-bearing pyrite.

i) Framboidal pyrites consist of spherical aggregates ~ 10 µm in diameter that are composed of nm to µm scale euhedral microcrystals. Many examples are overgrown, and/or infilled by later generations of pyrite, and may have an external pyrite halo (Fig. 3; ESM Fig. 6). Petrographic relationships indicate that this generation of pyrite is syn-sedimentary and predates significant burial. Framboids are often associated with organic-rich laminae, such as in OS244_128.

ii) Diagenetic pyrite consists of isolated euhedral pyrite crystals scattered within the matrix or concentrated along organic-rich laminae (ESM Fig. 6, inset a-b). Petrographic relationships indicate that this generation of pyrite most likely grew during sedimentary burial and lithification.

iii) Late pyrite may also be observed as up to 5 µm thick (generally 0.5–2 µm) coatings on framboidal and diagenetic pyrite (Fig. 3; ESM Fig. 6, inset b). The observed petrographic relationships indicate that this generation of pyrite formed after the lithification of the host rock. The late pyrite is often overgrown by realgar.

Many examples of pyrite display spongy or degraded outer boundaries, such as sample OS114_98 (Fig. 3). Pyrite in OS114_98 is highly corroded and is intensely altered by physical brecciation, silicification, dolomitization, and replacement by late pyrite and realgar (Fig. 3; ESM Figs. 1, 6). These features suggest that this sample may have been close to a conduit for mineralizing fluids.

The pyrites from the Conrad samples tended to have a finer As-rich rim (arsenian pyrite generally < 1µm), compared to those from the Osiris-Ibis-Sunrise samples, as well as having a more corroded appearance. Gold within the As-rich regions from Conrad was more difficult to find compared to Osiris-Ibis-Sunrise, partially due to the lower Au concentrations, as well as the finer nature of the rim (Fig. 3). The Osiris-Ibis-Sunrise pyrites generally showed larger, less altered rims, and tended to have a higher Au content than those from Conrad (Fig. 3). Arsenic and Au within the rims appeared, at the micrometer scale, to be relatively homogenous compared to those from Nevada (Gopon et al. 2019a). For full low and high resolution EPMA maps, see Penny (2020).

### FIB-SEM / APT

During FIB-SEM sample preparation for APT, a number of interesting features were noted. Within the core of the pyrite, we noted 1–2 µm sized inclusions of dolomite (Fig. 4). The rims, when viewed in cross section (cut by FIB-SEM), were uneven in size, and range between 0.5 µm and 2 µm wide. Also, visible within the cross sections of the rims were nanoscale voids, which give to the overgrowth pyrite a ‘spongy’ appearance (Fig. 3).

From the 34 total APT needle shaped specimens that were prepared, 8 successful APT experiments where conducted, with the rest of the samples disintegrating in the APT prior to a statistically meaningful number of ions being measured (our cutoff being 4 million ions). This 23.5% successful yield rate is slightly lower than that reported in other APT studies of sulfide minerals of between 25 and 50% (Fougerouse et al. 2016; Gopon et al. 2019a, 2022; Wu et al. 2021; Taylor et al. 2022; Atienza et al. 2023). However, none of these other studies analyzed sulfide minerals that contained voids, which are often noted to be the cause of premature fracturing of the sample. Six APT datasets were acquired from the Osiris-Ibis-Sunrise pyrite (3 each from the core and the rim), and 2 APT datasets were acquired from Conrad (1 from a pyrite framboid at the center of the ‘core’ and 1 from the ‘bulk’ core). No successful APT dataset was acquired from the Conrad pyrite rim, with all APT needles fracturing during the first few minutes of analysis. This can be attributed to the higher proportion of voids that were present in the Conrad pyrite rims compared to those from Osiris-Ibis-Sunrise (Fig. 4). APT mass spectra from each data set are shown in ESM Figs. 7–14.

**Fig. 5:**
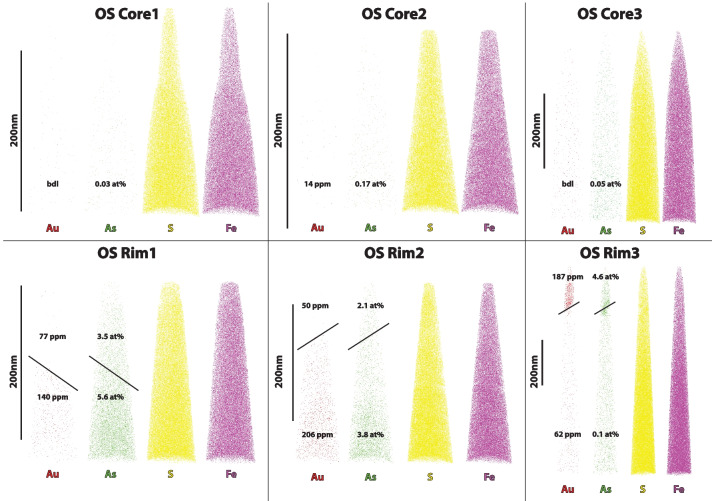
APT 3d volume reconstructions of pyrites from the Osiris-Ibis-Sunrise deposit. See Fig. 4a for approximate location of datasets within the pyrite. Atomic percent arsenic and gold are directly shown on the figure, see Table 1 for full details. Note: Conrad datasets are not shown as no successful data was acquired from the rim

**Fig. 6:**
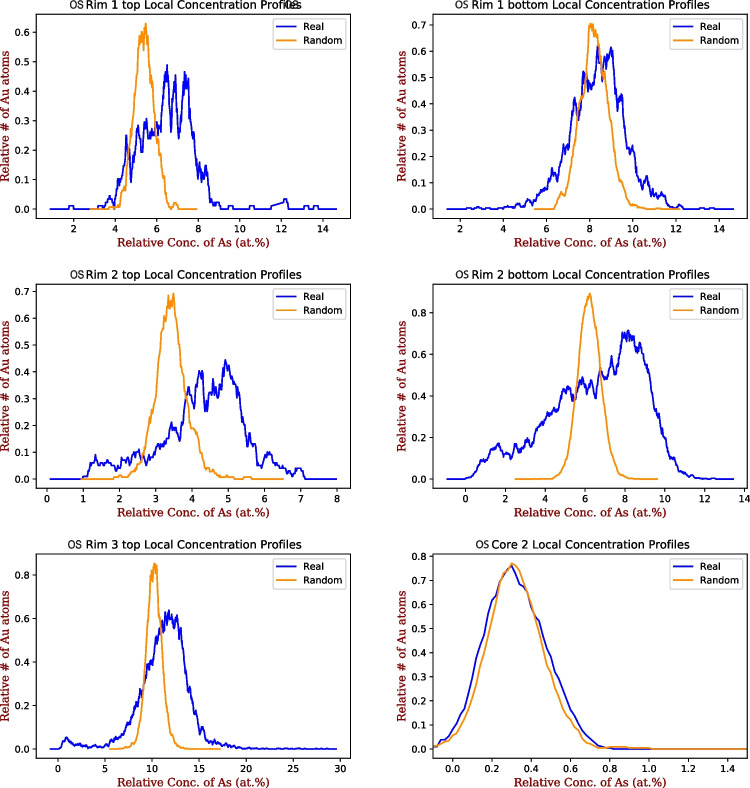
As/Au spatial distribution maps from the Osiris-Ibis-Sunrise pyrite datasets (sample OS244_128), split up between the high and low gold regions for OS Rim1,2; OS Rim3 shows only the gold rich region; and the core sample with minor gold is shown (OS Core 2). The data is plotted as the relative number of gold atoms (Y-axis; normalized to 1) that have a given (relative) concentration of As in a 3nm sphere surrounding them (X-Axis)

The rim datasets from Osiris-Ibis-Sunrise show a nanoscale zonation with varying Au and As contents within each sample (Fig. 5). The zones with higher As contents tend to have higher Au contents (Fig. 5, Table 1). Within these zones, Au is homogenously distributed, as visualized in Fig. 5, and this was confirmed using the cluster search function in the IVAS (v.3.8.8 software; Penny 2020). When the 3-Depict software was applied and correlation plots for Au/As generated, a shift to higher As concentrations was noted in ‘real’ compared to the ‘randomized’ data in the Au rich zones, and no shift was noted in the Au-poor zones (Fig. 6). For all Au-As containing zones, there is a shift in the real curve to the right (i.e. higher As) compared to the randomized data (i.e. approximation of matrix distribution). This is to say that in all rim datasets, and Au-rich sub-volumes of datasets, there is an increased As concentration surrounding the Au atoms compared to what would be expected in a matrix exhibiting a random distribution of As atoms.

**Table 1:**
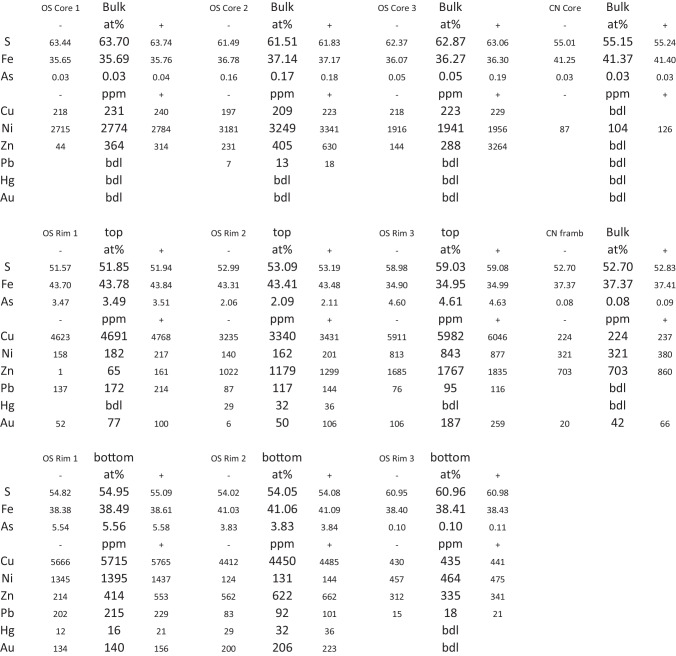
APT data from the Osiris-Ibis-Sunrise (OS) and Conrad (CN) deposits, Yukon. Data is reported as atomic % and atomic ppm with low (-) and high (+) error bars as calculated from the uncertainty of the overlap correction

**Fig. 7:**
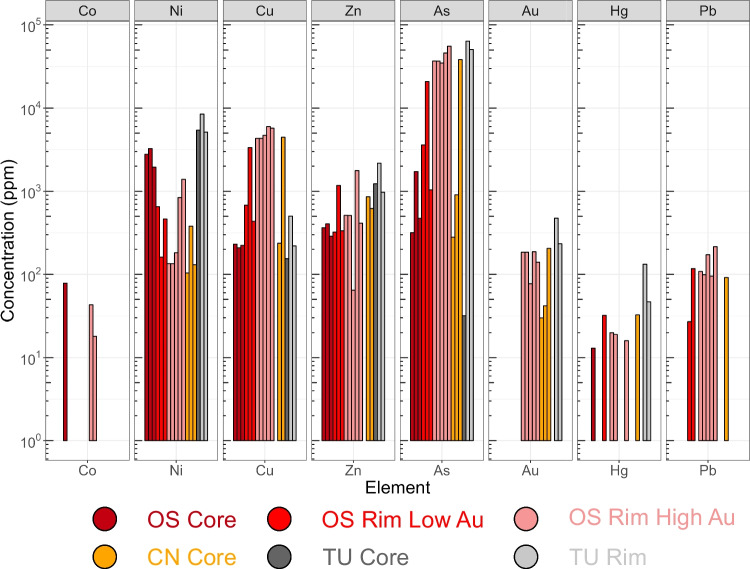
Bar chart of APT data from gold bearing and barren pyrite from the Yukon and Nevadan Carlin-type gold deposits. BDL values are not shown on the plot

When the trace element data is compared between the core and rims of the Yukon pyrite, there is evidence for a near absence of Au and As in the core, as well as a depletion in other trace metal contents (Table 1; Fig. 7). The highest concentrations of nickel (3249 ppm) is found in the core of the Osiris-Ibis-Sunrise pyrite. The framboidal core of the Conrad sample contains slightly elevated Ni compared to the Au rich regions, while the non-framboidal core contains less Ni and generally less trace metals than the framboidal core (Table 1; Fig. 7). The high Au rim datasets contain more of the heavy trace metals (Pb, Hg) compared to the low Au rim datasets. The transition metals are more variable in the high and low Au rim datasets, but the high Au portions of the datasets have higher transition metal contents relative to the low Au portion. The re-processed data from the Turquoise Ridge CTG deposit in Nevada (Fig. 7; labeled TU Rim, TU Core) contain higher As and Au concentrations compared to the Yukon samples, as well as generally higher transition metal concentrations, except for Co and Cu. Mercury is highest in the Nevadan Au containing rim datasets (TU rim), while also being devoid of Pb.

## Discussion

When comparing the Carlin-type deposits in the Nadaleen Trend with those in Nevada, a number of similarities have been noted (Arehart et al. 2013; Pinet and Sack 2019; Pinet et al. 2022c). These relate primarily to similar depositional environments of the host rocks as well as tectonic preparation prior to mineralization, as well as similar alteration, paragenesis and metal content. In both cases, the age of mineralization is > 270 Ma younger than the host-rocks (mineralization age: 74–71 Ma for Yukon and 42–36 Ma for Nevada; Arehart et al. 2003; Hofstra et al. 1999; Pinet et al. 2022b). However, the Nevadan ore mineralization has been linked to extensional magmatism (Cline et al. 2005; Muntean et al. 2011; Holley et al. 2019) whereas it is linked to a regionally transpressional period with only minor magmatism in the Nadaleen Trend (Pinet et al. 2016; Steiner et al. 2017). It should be noted that the absence of a direct link to magmatism was debated for the Nevadan deposits over many decades (Muntean et al. 2011), and the possibility that the Nadaleen CTG deposits are related to buried intrusive bodies must be kept in mind (Pinet et al. 2020). The sulfur isotopic ratio of the Au containing overgrowths might provide some indication of the source of fluids (i.e. magmatic vs orogenic), but the small size of the overgrowths has made detection challenging. S-isotopic analysis of APT data has been demonstrated by Gopon et al. (2022), and this will be investigated in a follow on paper.

### Sedimentary / detrital pyrites and their host rocks

The host rocks of the Yukon deposits are mainly lime mudstones, intraclastic packstones, and floatstones with subordinate calcareous siltstones and mafic dykes. The succession was deposited in a variety of offshore environments, probably below fair weather and storm wave base, in slope, to base of slope settings (Moynihan et al. 2019). In both the Yukon and Nevadan CTG deposits, Au-rich overgrowths occur on Au-As-barren pyrite cores, which in both settings have been interpreted as sedimentary/diagenetic in origin (Cline et al. 2005; Large et al. 2011; Pinet and Sack 2019). The pyrite cores in Nevada have a generally more euhedral appearance, although a fair amount of textural and size variability has been noted (Barker et al. 2009; Longo et al. 2009; Gopon et al. 2019a). The barren pyrites from the Nadaleen Trend are variable in shape, with pyrite forms ranging from spherical to pyrite framboids. Sizes of pyrites dominantly range from one to a few tens of µm with rare (usually diagenetic) pyrite greater than 100 µm. The smallest (1–3 µm) pyrites are the most abundant by number, and often (but not always) exhibit a core/rim structure with the cores being < a few hundred nm. The lack of a visible core in some of the smallest pyrites might be attributed to spatial resolution of our SEM, as well as the 3-D effect which might be hiding a small core in the third dimension of our thin section.

The cores of the pyrites from the Yukon and Nevadan CTG deposits are quite distinct in their trace element chemistry, with the Nevadan ores generally being enriched in the transition metals investigated compared to those from the Yukon (Fig. 7). The relative abundances of the trace elements within the pyrite cores are similar, however, with As > Ni > Zn > Cu. The absolute amounts of the trace elements probably reflects the original metal content of the sediment rather than any differences in the redox state of the sediment or the temperature of the water column (Large et al. 2014; Gregory et al. 2015). The absence of detectable Cu and Zn in the cores of the Conrad pyrite, especially when compared to the microcrystal at the center of the same pyrite, is notable. This is considered to represent either changing redox conditions, or the differences in the medium (i.e. sea water or sediment) in which the framboid might have grown. It should be noted that high Cu has been noted in modern pyrite framboids (Atienza et al. 2023), which might point to important differences between in depositional setting and/or ocean evolution. However, the difficulty of accurately quantifying Cu with APT, due to the overlap on of S_2_^+^ on the Cu^+^ at the 64 Da peak, means that caution interpreting this data is required, especially when using the ‘standard’ overlap algorithm in IVAS (Gopon et al. 2022).

Yukon host rocks are generally rich in organic matter, as supported by the observation of organic rich laminae (black shales) in OS244_128 and by the total organic content (TOC) determined through programmed pyrolysis analyses (Pinet et al. 2022a). In addition to supplying elements such as S and P to the sediment pile, organic matter has been implicated in the formation of pyritic ores in similar settings (Williams 1978; Hayward et al. 2021).

The timing of pyrite growth within the host sediments can be constrained using petrographic relationships. Framboidal pyrite formed prior to burial and compaction, probably during sedimentation when sediment pore waters were still exchanging with the overlying water column. It seems likely, from the displacement of sedimentary laminae around framboids, that their formation took place prior to burial and compaction. The diagenetic pyrite fills inter-grain pore space, particularly in organic rich laminae, which indicates that this generation of pyrite grew while pore spaces were open and when pore fluids were saturated with respect to pyrite. However, the isolated nature of the diagenetic pyrite grains in pores indicates that pyrite saturation was localized (ESM Fig. 6), suggesting that that pore fluids were disconnected from open marine waters by this stage. Thus, the diagenetic pyrite grew during burial, compaction, and lithification of the host sediment. The late (ore stage) pyrite coats the earlier generations of pyrite, and infills residual post compaction pore spaces and must, therefore, have formed after burial and compaction. Additionally, the late pyrite coats the walls of fractures and micro faults, which have offset and brecciated the host rock. Realgar is associated with the late pyrite, and fills post compaction pore spaces, fractures, and micro-faults. This realgar commonly displays zonation indicative of multiple phases of growth, which may indicate repeated pulses of mineralizing fluids. These petrographic features clearly demonstrate that the late Au bearing pyrite grew after burial, compaction, and lithification of the host rock. Simply stated, the mineralization fits with an age much later than the sediment deposition.

### Textural and geochemical evidence from the micrometer to nanometer scale

The most obvious differences between the Au containing arsenian pyrite overgrowths from Nevada and the Yukon, are that those in Nevada are much larger (5–10 µm vs < 2 µm) and that the Yukon Au containing pyrite contains less As and less Au. The Yukon Au rich pyrite also has a spongy appearance, due to the presence of numerous void spaces, not noted in Nevada (Palenik et al. 2004; Gopon et al. 2019a). However, despite the smaller size and lower Au content of the Yukon CTG pyrite rims, the ore grades in the Yukon are as high as many Nevadan CTG deposits, with 23.7% of ore samples (> 0.5 g/t; N = 2582) grading > 5 g/t in the Yukon (ATAC-Resources 2021). Otherwise, at the micro- and nano-scale, all other features of the Yukon CTG are comparable to those in Nevada. The Au-rich rims in the Yukon CTG are similarly zoned in their As and Au contents, although in the Yukon samples this is a nanoscale zoning, while in the Nevadan Au rich rims zoning is at a micrometer scale. Similar trends in As and Au concentrations are noted in the pyrite rims, with higher Au concentrations being linked with elevated As, whether it be in the micrometer zoning in Nevada or the nanometer zoning in the Yukon pyrite (Fig. 5; Table 1; Gopon et al. 2019a). The smaller rims, and smaller zones within those rims, in Yukon could be related to shorter ore pulses or to the smaller grain sizes in the Yukon that allowed for more nucleation sites for hydrothermal pyrite, thereby diluting the size of the overgrowths on individual pyrites.

The trace metal signatures measured in the Au containing rims are comparable between Nevadan and the Yukon CTG deposits, with the exception of three metals. Copper and lead are found in significantly higher concentrations in the Yukon Au-bearing pyrite than in Nevada, while the Nevadan Au bearing pyrites, and the Nevadan deposits in general, are enriched in mercury (Fig. 7; Harris and Radtke 1976). As the Yukon and Nevadan CTG are generally devoid of stoichiometric copper or lead sulfides (i.e. chalcopyrite and galena), all of the Cu ought to end up in the dominant sulfide phase (i.e. pyrite) in both these deposits. The large differences in Cu between Nevadan and Yukon pyrite might therefore represent real differences in the copper and lead concentrations of the fluids. The elevated Cu in the Yukon arsenian pyrite might be an indicator of a magmatic fluid source component, whereas the lower Cu and Pb and elevated Hg in Nevadan arsenian pyrite may reflect either a different type of fluid source or the more distal part of a magmatic system.

### Insights from atomic scale geochemical analysis

**Fig. 8:**
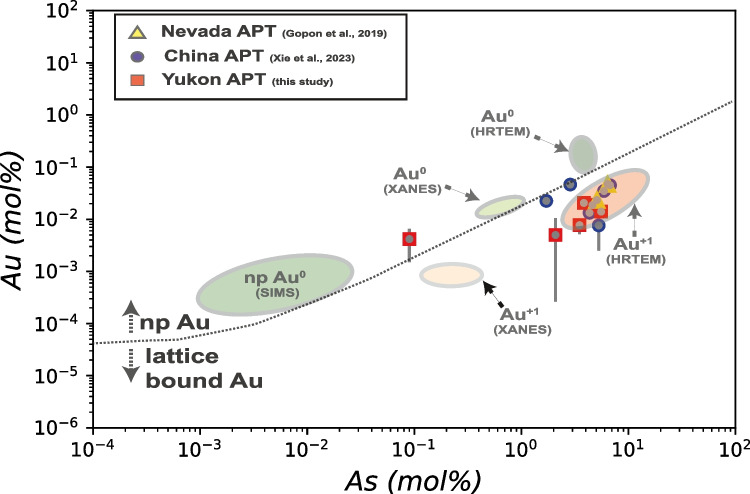
As vs Au values from APT data from this study plotted onto a simplified version of the As/Au plot from Reich et al. (2005). For comparison APT data from Nevada (Turquoise Ridge/Getchell Deposit; reprocessed from original Gopon et al. (2019a) and China (Shuiyindong and Lannigou Deposits; Xie et al. 2024). Bdl values are not included on this plot. Note that all datapoints fall, within error, below the dotted line defined by Reich (2005) as the solubility limit of Au in arsenian-pyrite

Gold in the Nadaleen pyrite rims, has been noted to be homogenously distributed (Fig. 5), similar to the occurrence at Turquoise Ridge in Nevada. The increased concentrations of As surrounding Au atoms (Fig. 6) noted in this work is also similar to that reported in Nevada (Gopon et al. 2019a), as well as in CTG deposits in SW-China (Xie et al. 2024). This implies that both lattice-bound Au and the incorporation of Au assisted by As, seem to be important components of CTG mineralization. Furthermore, when looking at the ratio of Au/As of Au containing pyrite in Nevada and Yukon (Fig. 8), the data (within error) never plots above the ~ 1:200 Au/As solubility limits determined by Reich et al. (2005) suggesting that this is a common phenomenon for CTG mineralization.

Homogenously distributed Au, As enrichment surrounding Au atoms, and the ratio of Au to As not exceeding a ~ 1:200 ratio are common features in both the Yukon and Nevadan APT data, strongly suggesting that there is a common mechanism at work which allows for the incorporation of appreciable amounts of Au into pyrite (Reich et al. 2005; Deditius et al. 2014; Kusebacuh et al. 2019). If we combine these three main observations from the CTG APT data, with the observations of Reich et al. (2005), the coupled behavior of Au-As in the pyrite growth experiments of Kusebauch et al. (2019), and the hypothesized incorporation mechanism of Au into pyrite from Fleet and Mumin (1997), we can hypothesize a commonly applicable model for Au incorporating into pyrite:

The two main elements in pyrite, Fe and S, generally occur in the 2^+^ and 2^−^ charge states, respectively. As pyrite contains two S atoms, this does not charge balance; however, the two S ions share a covalent bond, which charge neutralizes a -1 charge from each S ion. In essence, a S in pyrite has a 2^−^ charge, but since it shares an (S–S) bond, S_2_ also has a 2^−^ net charge. Gold is a larger ion than Fe, and generally occurs as Au^+^. Therefore, Au has no simple substitution mechanisms by which it can incorporate itself into the cubic pyrite structure. However, As can occur as a range of cations and anions (3^+^, 2^+^, 1^−^, 2^−^, or 3^−^) and can therefore substitute both for Fe and S in the pyrite structure (Fleet and Mumin 1997; Pokrovsky et al. 2021). Evidence suggests that As more commonly replaces for S in this deposit (Fig. 3) as well as in other CTG deposits, likely in the 2^−^ charge state (Reich et al. 2005; Deditius et al. 2014; Gopon et al. 2019a; Xie et al. 2024). In the case of As – S substitution in pyrite, this usually occurs as a soft Lewis base (ex. AsS, As_2_; Fleet and Mumin 1997). Combining this information leads us to a potential new model for the incorporation of Au and As into pyrite.

The differences in radii between S and As, as well as the slight charge imbalance that exists between these elements, means that both a point defect site, as well as a small local negative charge (~ 0.005^−^) might be created by the substitution of As for sulfur. The more As replaces S on a local level, the larger the defect, and the larger the charge imbalance would become. At the point of ~ 200 As ions replacing for S in a subdomain of our pyrite a local 1^−^ charge would be created, as well as a large enough defect, which would allow the large Au^+^ to fit and balance the 1^−^ charge (Gopon et al. 2019a; Xie et al. 2024). It would then be a local ‘cloud’ of As that allows for the incorporation of Au into pyrite, rather than a more simple substitution mechanism.

Such a ‘cloud’ of enriched As surrounding Au ions can indeed be visualized in the As/Au atomic spatial distribution maps (Fig. 6), by the shift to higher As concentrations of the actual data compared to the randomized data (i.e. approximation of homogeneous matrix). This is strong evidence that a more complex substitution mechanism, such as described above, is at play. This As ‘cloud,’ as well as the correlation between high Au and As at the micro- and nano-meter scales, was also noted in the Nevadan samples (Fig. 6; Gopon et al. 2019a), which implies that this ‘As cloud’ substitution model is an important aspect of CTG mineralization.

Alternative explanations for the incorporation of Au in pyrite do exist and must be considered (Palenik et al. 2004; Barker et al. 2009; Fougerouse et al. 2016; Pokrovski et al. 2021; Wu et al. 2021). The strongest alternative hypothesis is that the rapid growth of pyrite in a hydrothermal setting means that Au (either as individual Au^+^ ions or nanoparticles of Au^0^) can be become incorporated into the imperfect rapidly growing crystal, without it being properly incorporated into the crystal structure. Of the proposed alternative explanations, some involve As in other ways (Pokrovski et al. 2021) while others do not have As playing any role in the Au incorporation (Wu et al. 2021). A potentially important consideration is the role of temperature on the solubility of Au and As in pyrite (Deditius et al. 2014). There does appear to be a decrease in Au-As contents in pyrite with increasing temperatures, but the relative ~ 200:1 As:Au trend is unchanged at high vs. low temperature deposits (Deditius et al. 2014).

The strong correlation between high Au and high As (regardless of temperature) on the deposit- to nano-scale, does suggest that there is some link between As and Au, and that a totally As free explanation is unreasonable. Recent experimental studies have shown that Au nanoparticles are attracted to the surface of arsenian-pyrite (Nie et al. 2023), which offers a potentially intriguing alternative explanation that combines rapid growth while still retaining a role for As. If Au, as nanoparticles or Au^+^ ions, is attracted to the surface or As-rich pyrite it might be ‘swallowed’ by the rapidly advancing imperfect growth surface, without As necessarily helping during Au incorporation. While this does explain the correlation between Au/As, it fails to account for the cloud of As that has been observed in all APT data of CTG pyrites (Xie et al. 2024; Gopon et al. 2019a). If we, however, combine our incorporation mechanism with the work of Nie et al. (2023), it gives two complimentary mechanisms whereby to enrich Au into arsenian-pyrite. In this combined model, As is both a pump that attracts Au to the pyrite growth surface, and the substitution of As for S (as described above) in the pyrite lattice allows for appreciable amounts of Au to be incorporated.

While the alternative explanations of Fougerouse et al. (2016), Wu et al. (2021), Palenik et al. (2004) fail to account for all of our findings, we must point out that our findings do not account for the, potentially rare, occurrences of Au nanoparticles in CTG pyrite. While it is debated how common these nanoparticles actually are in each deposit, they have been noted in Nevada as well as CTG deposits in SW China (Palenik et al. 2004; Liang et al. 2021). Two possible explanations for these nanoparticles exist: 1) they are nanoparticles of Au that were adsorbed to the growth surface and incorporated during rapid growth, or 2) they form during solid-state remobilization of Au during later changes in the P/T/pH/fO_2_ during subsequent metamorphism. Without experiments to recreate these processes it is difficult to ascertain which of these competing hypotheses is most likely. However, it should be noted that at least one orogenic Au deposit (Flatschach Cu-Au deposit, Styria, Austria) with Au containing arsenian-pyrite shows microstructural evidence suggesting that solid state remobilization during metamorphic overprint leads to formation of Au particles in pyrite (Niederl et al. 2022). This remobilization of trace elements in sulfide minerals, has also been observed in Co containing sphalerite in Namibia (Bertrandsson Erlandsson et al. 2023) and The containing pyrite in Fiji (Börner et al. 2023), which not only adds credence to our Au re-mobilization model, but also shows that solid-state remobilization during metamorphism is a potentially important process in secondary enrichment of trace elements. Beyond this petrographic evidence from other deposits, we point out that in all the carefully selected pyrites from three ‘representative’ CTG deposits in Nevada and Yukon, the Au is structurally bound. Furthermore, in a recently published work from two representative CTG deposits in SW China (Shuiyindong and Lannigou deposits), APT analyses confirmed that Au is structurally bound (Xie et al. 2024). Interestingly, this work found identical features in the Chinese CTG deposits to those described in this manuscript for Nevadan and Yukon deposits, i.e. Au/As correlation at the micro- to atomic-scale, As enriched ‘clouds’ surrounding Au ions, ~ 1:200 ratio of Au to As (Fig. 8; Xie et al. 2024). Based on this evidence, it seems that even if Au nanoparticles are incorporated during the original mineralization, it is not the primary way that Au is hosted in CTG deposits.

In summary, our observations suggest that Au in Yukon and Nevada, and in fact all CTG deposits thus far studied with APT (Gopon et al. 2019a; Xie et al. 2024): 1) primarily occurs as structurally bound Au^+^; 2) is surrounded by an enriched region of As; 3) is incorporated into arsenian-pyrite by As-S substitution which imparts a lattice defect and charge imbalance that Au^+^ can occupy in and charge balance. This ‘cloud’ of As that surrounds individual Au atoms, and that the micro-and nano-scale ratios of As:Au follows the ~ 200:1 solubility trend defined by Reich et al. (2005), suggests that for every 200 As ions the incorporation of one atom of Au can be accomplished. Given the similarities at the micro-, nano-, and atomic-scales of the CTG deposits thus far investigated with APT (Turquoise Ridge/Getchell, Osiris-Ibis-Sunrise, Conrad, Shuiyindong, and Lannigou), it seems likely that our observations and envisaged Au trapping and incorporation mechanisms have wide-ranging applicability. The presence of As in these systems is therefore an integral component of the ore-forming system.

## Conclusion

1. Atom Probe Tomography (APT) shows that Au, in the CTG deposits investigated in Nevada and Yukon, is lattice bound (Fig. 5) and is linked at the micrometer to atomic scale with As (Fig. 6). This suggests that the imperfect substitution of As for S, which imparts lattice defects and charge imbalances, is essential in allowing pyrite to host Au^+^, with roughly 200 As ions allowing 1 Au ion to be incorporated.
2. Gold-rich overgrowths in Yukon pyrites (Fig. 3) are complexly zoned, as in Nevada, but at the nanoscale, rather than the micrometer scale seen in Nevada. Yukon Au rich pyrite tends to have a spongy appearance compared to the Nevadan counterparts. Yukon pyrite rims are much smaller, and tend to have a more altered appearance, compared to those in Nevada.
3. Gold and As concentrations (Fig. 7) in Osiris-Ibis-Sunrise and Conrad (Yukon) are slightly lower in the As-rich overgrowths compared to Turquoise Ridge (Nevada). Transition metal concentrations in the sedimentary cores of the Nevadan pyrites are generally higher than those in the Yukon, with the exception of Ni and Pb, while the Au-rich growth zones in Nevadan pyrites have higher transition metal concentrations compared to those from the Yukon, with the exception of Cu. Mercury is found in much higher concentrations in Nevadan Au-rich pyrite zones compared to the Yukon, while the Yukon Au rich pyrite zones tend to have appreciable lead concentrations.

## Supplementary Information

Below is the link to the electronic supplementary material.

Supplementary file1 (PDF 51102 KB)
