# Supplementary material for: Revealing Yukon’s hidden treasure: an atomic-scale investigation of Carlin-type gold mineralization in the Nadaleen Trend, Canada
Source: Miner Depos. 2024 Oct 30;60(5):937–53. doi: 10.1007/s00126-024-01325-9 (PMC12065730; doi:10.1007/s00126-024-01325-9)
Supplement: Supplementary file 1 — Supplementary file1 (PDF 51102 KB) [file 126_2024_1325_MOESM1_ESM.pdf]

ESM Figure 1: Sample OS114_98 location, core photographs, overview thin section images, and drillhole data from OS114 showing Au, Ag, Hg, Ca, and Mg concentrations downhole.

Sample location:
Deposit: CONRAD
Drill hole: OS-12-114
Easting (NAD83-zone 8): 630404
Northing (NAD83-zone 8): 7112505
Dip: 89.4°
Sample depth: 97.42 m

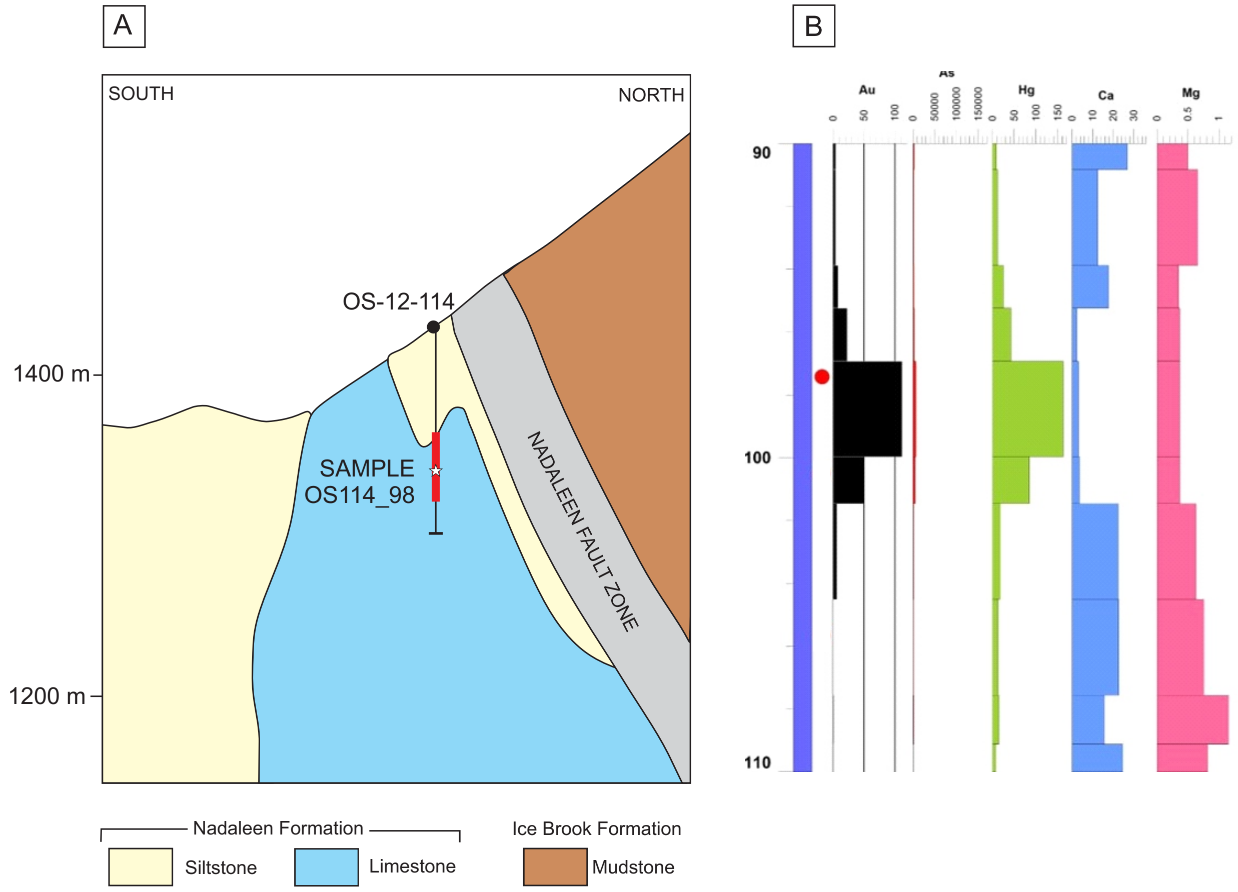

A) Cross section of the Conrad deposit showing the location of the sample OS114_98 (modified from ATAC Resources). B) Au, As, Hg, Ca and Mg content of the drill hole OS-12-114 between 90 and 110 m depth. Geochemical analyses from ATAC Resources database.

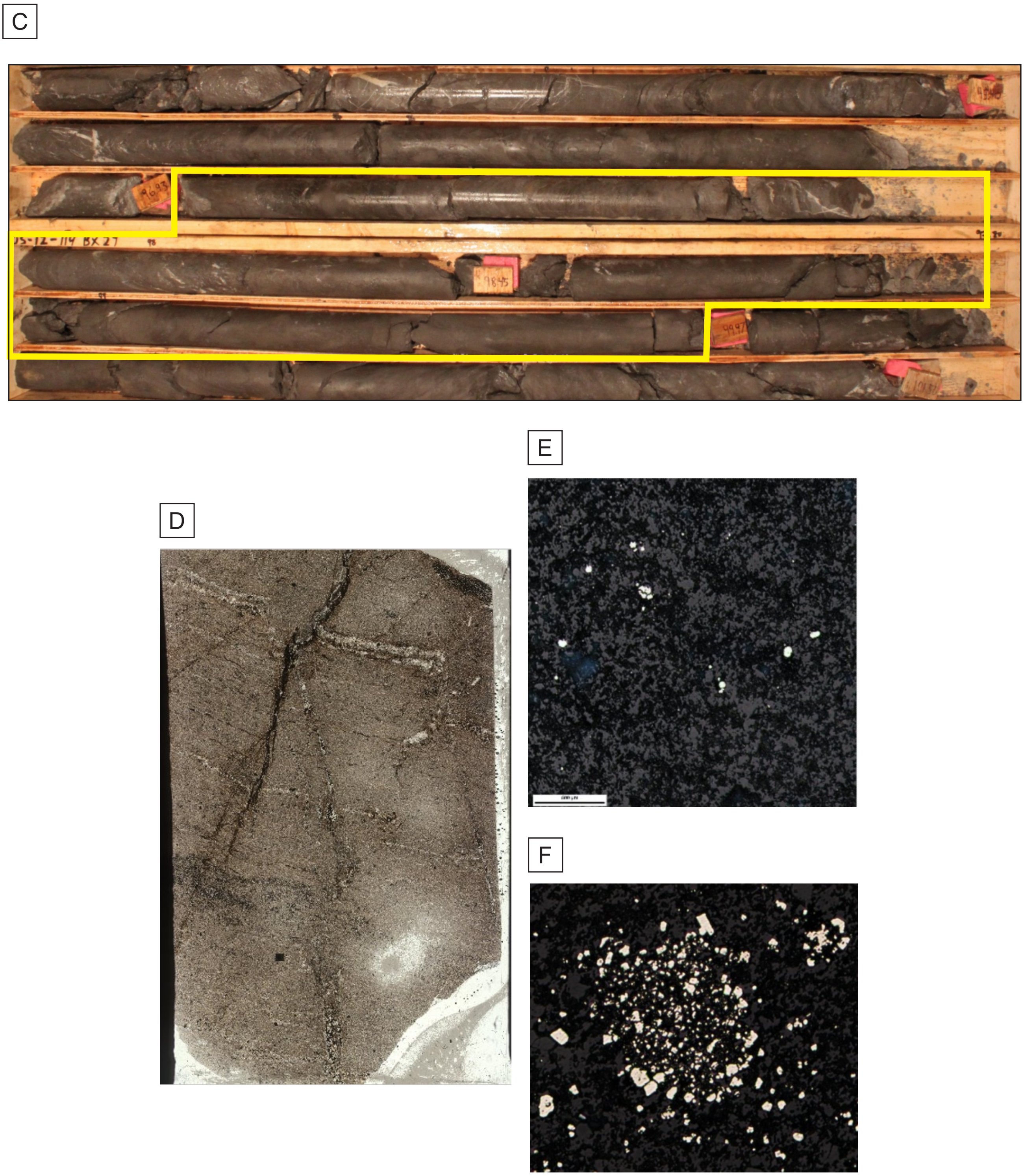

C) Picture of the core interval including sample OS114_98. The yellow box corresponds to a 3.04 m interval that yielded 111.5 g/t Au. D) Thin-section of sample OS114_98. E) and F) Microphotographs of sample OS114_98 showing disseminated pyrite (E) and a cluster of pyrite grains (F).

HoleName	OS -12-114
DepthFr	96.93
DepthTo	99.97
Interval	3.04
Ag	0.07
Al	5.68
As	6760
Au	111.5
Ba	260
Be	0.84
Bi	0.28
Ca	3.18
Cd	1.2
Ce	64.5
Co	23.2
Cr	57
Cs	12.45
Cu	48.4
Fe	5.95
Ga	17.7
Ge	0.22
Hf	2.7
Hg	162.5
In	0.057
K	2.58
La	35.6
Li	12.1
Mg	0.37
Mn	341
Mo	1.23
Na	0.02
Nb	8.6
Ni	34.6
P	520
Pb	19.2
Rb	117
Re	0.004
S	6.63
Sb	305
Sc	8
Se	2
Sn	2.4

ESM Figure 2: Geochemical analysis of drill hole OS-12-114 between 96.93 and 99.97 m. From ATAC Resources database.

ESM Figure 3: Sample OS244_128 location, cross section, core photographs, and overview thin section images.

Sample location:
Deposit: OSIRIS
Drill hole: OS-17-244
Easting (NAD83-zone 8): 629379
Northing (NAD83-zone 3): 7112459
Dip: ca. -45°
Sample depth: 128 m

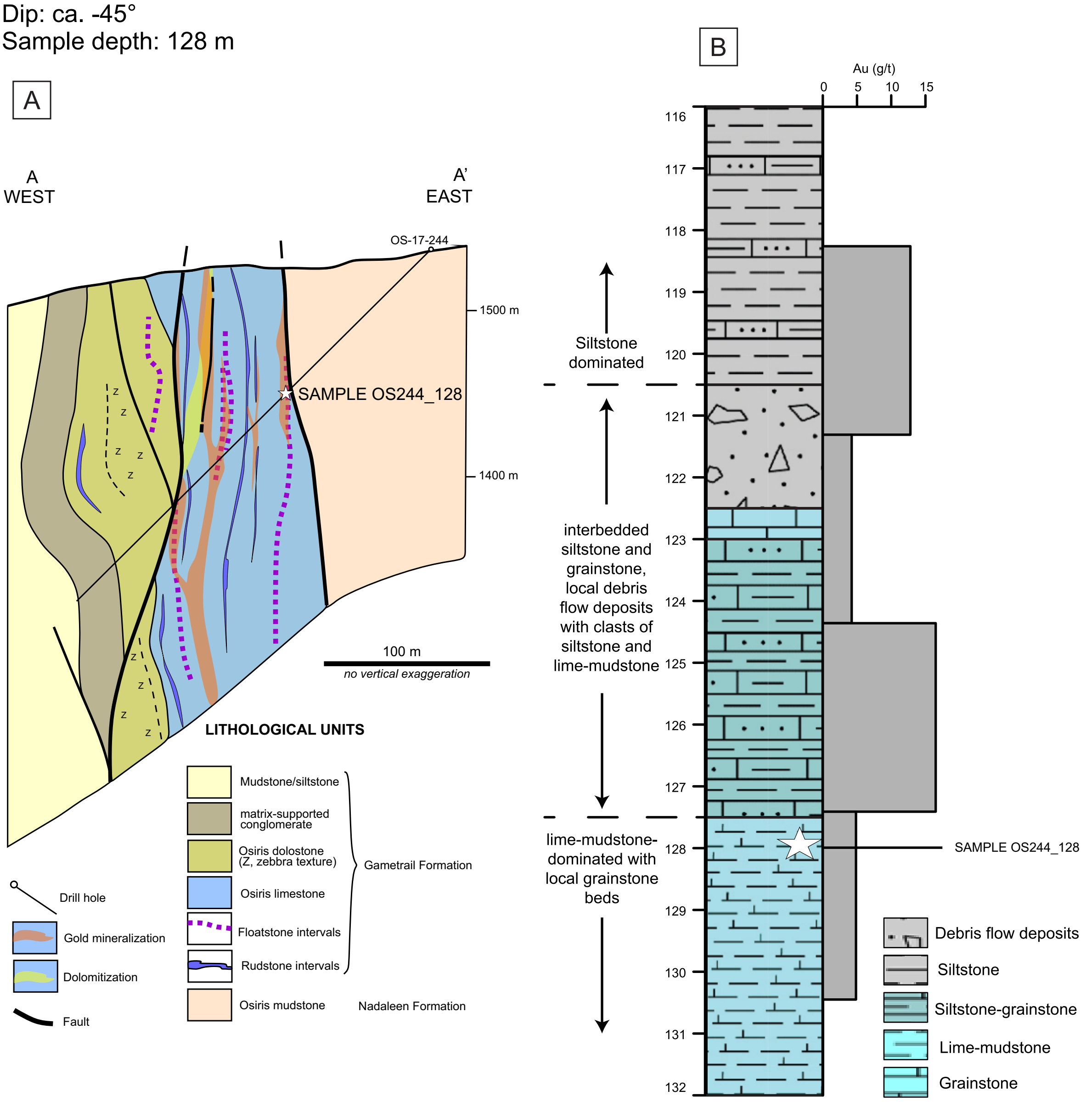

A) Cross-section of the Osiris deposit showing the location of sample OS244_128. B) Log of drillhole OS-17-244 from 116 to 132 m (modified from Pinet and Sack, 2019).

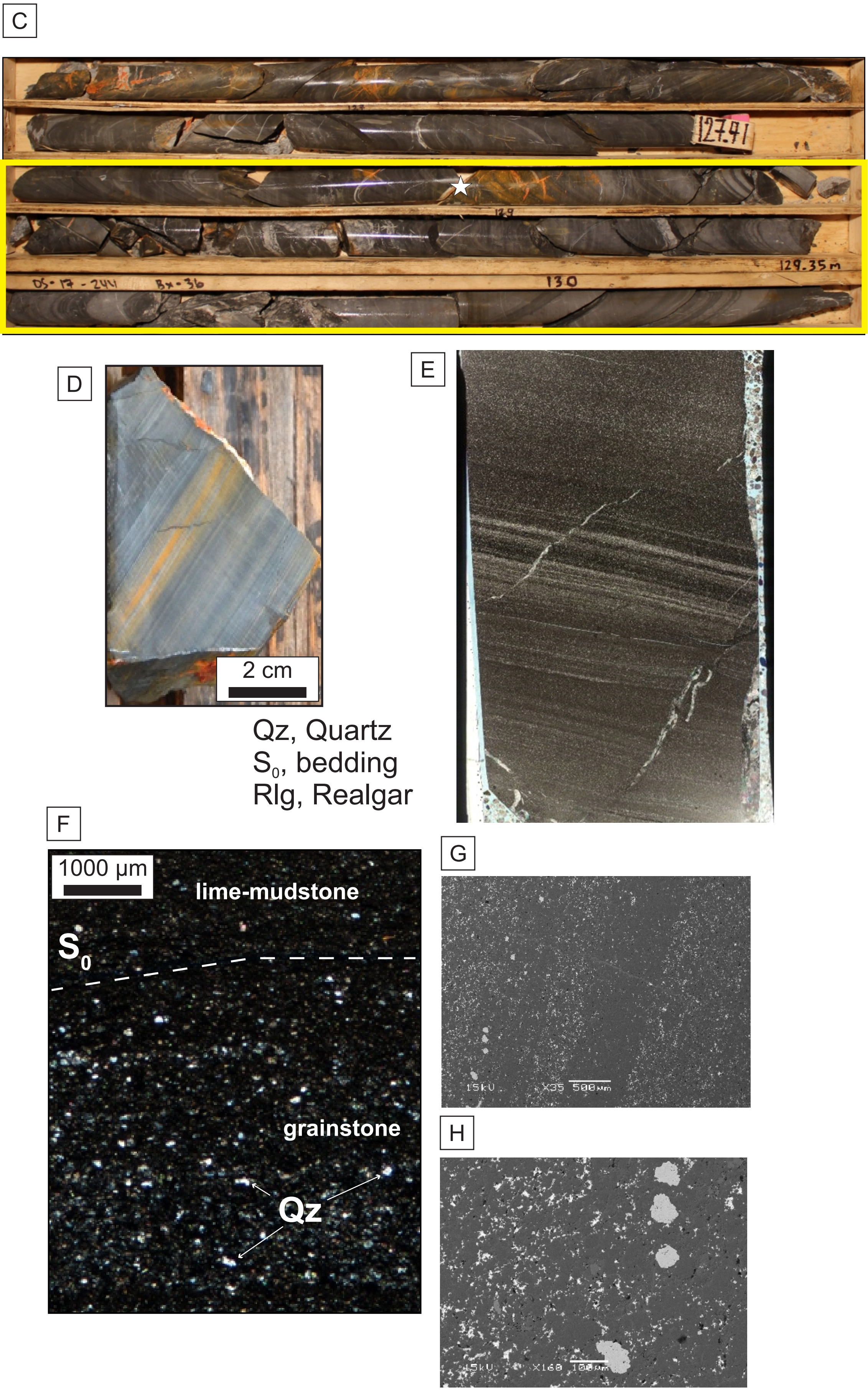

C) Core picture of drill hole OS-17-244. The interval that yielded 4.85 g/t Au over 3.04 m is indicated by a yellow box. The star indicate the location of sample. D) to H) Pictures of sample OS_128 at various scales

HoleName	OS -17-244
Zone	Osiris
DepthFr	127.41
DepthTo	130.45
Interval	3.04
Ag	1.13
Al	2.82
As	5440
Au	4.85
Ba	130
Be	0.92
Bi	0.07
Ca	16.65
Cd	0.09
Ce	34.5
Co	4.9
Cr	33
Cs	6.18
Cu	20.7
Fe	1.46
Ga	7.8
Ge	0.08
Hf	1.4
Hg	1.865
In	0.024
K	1.49
La	17.4
Li	12
Mg	3.44
Mn	981
Mo	0.44
Na	0.02
Nb	4.9
Ni	11.5
P	300
Pb	5.8
Rb	60

ESM Figure 4: Geochemical analysis of drill hole OS-17-244 between 127.41 and 130.45 m. From ATAC Resources database.

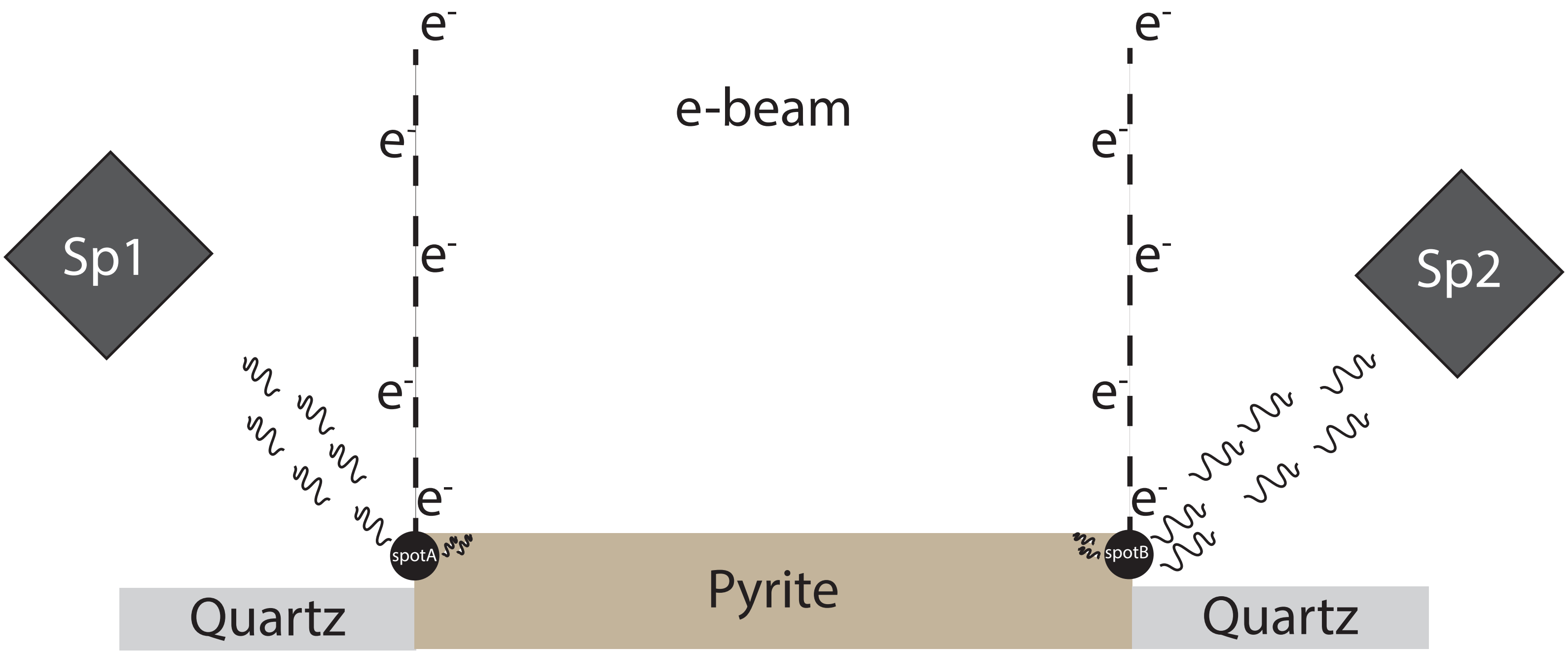

ESM Figure 5: Cartoon showing the effects of edge effects on the signal seen by WDS detectors. In the example of spotA, Sp2 would be shadowed by the sample and receive less signal, as opposed to Sp1 which has no barrier between it and the detector and would much higher count rates compared to Sp2 as a result. The opposite would be true at spotB, and Sp1 would have less signal compared to Sp2

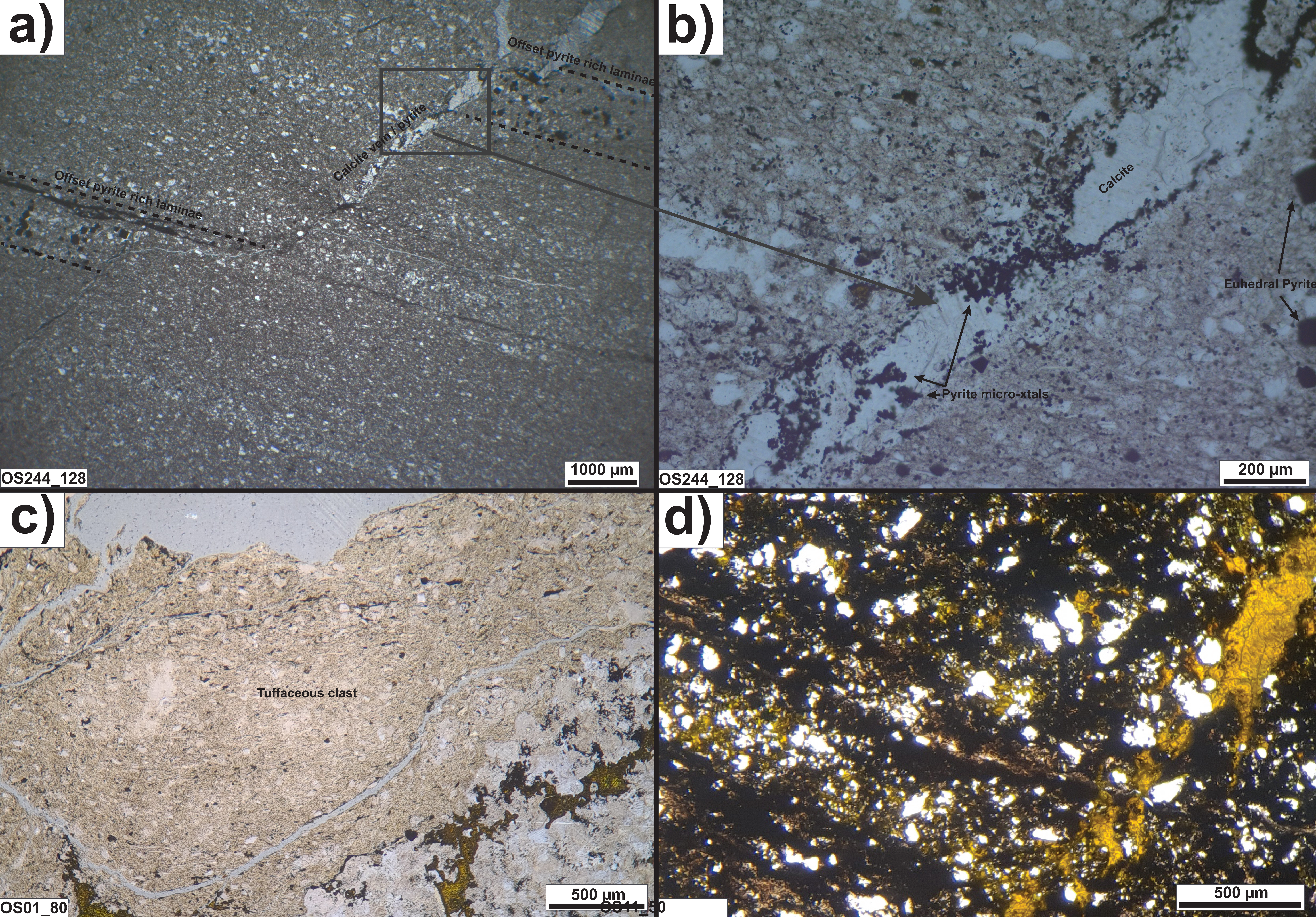

ESM Figure 6: Photomicrographs showing selected sedimentary and hydrothermal microstructures. a) shows an offset pyrite rich laminae; b) a zoom in of the calcite veins from inset (a) showing its association with numerous micrometer scale pyrites; c) a tuffaceous clast; and d) late realgar following existing vein structures

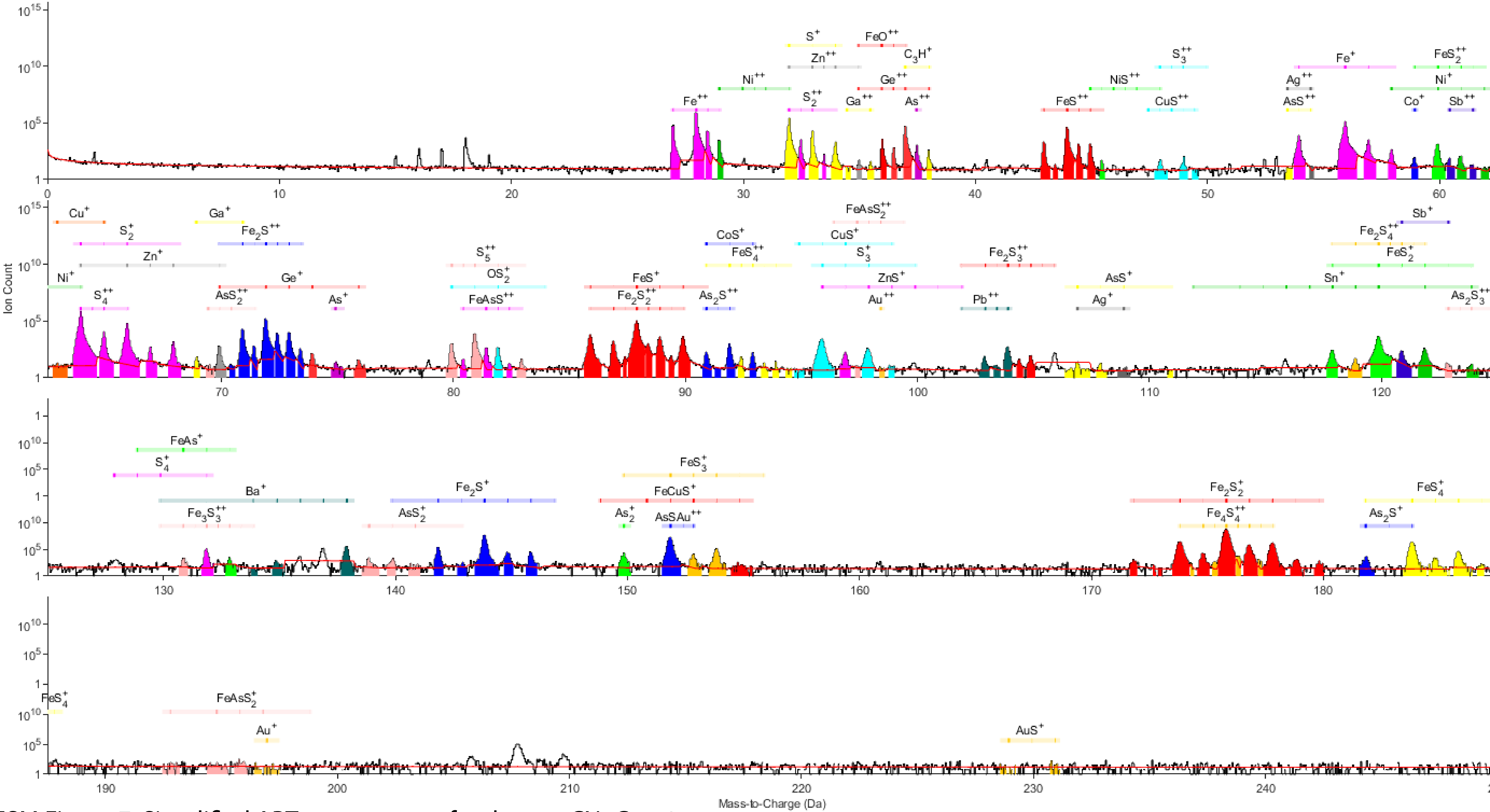

ESM Figure 7: Simplified APT mass spectra for dataset CN_Core1.

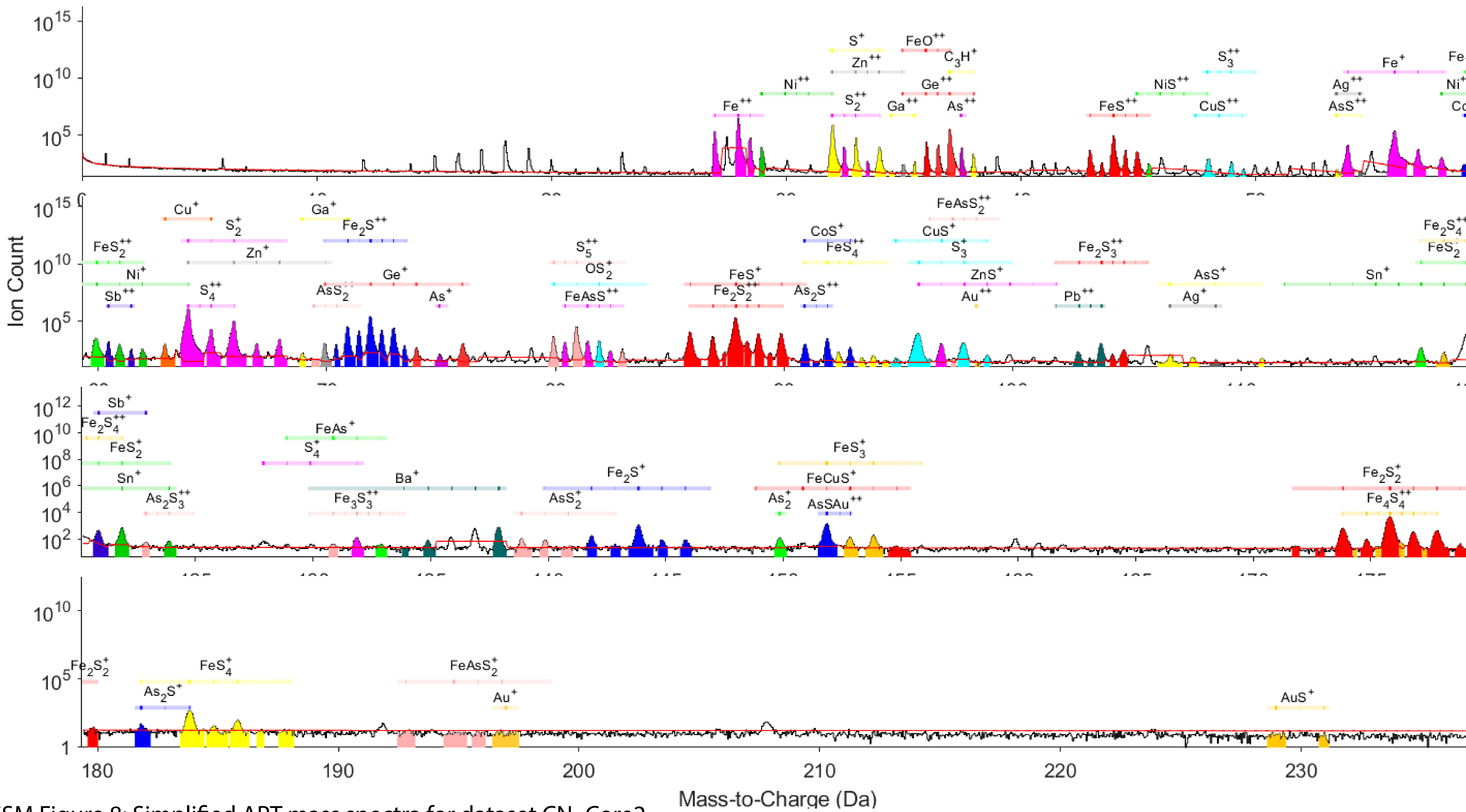

ESM Figure 8: Simplified APT mass spectra for dataset CN_Core2.

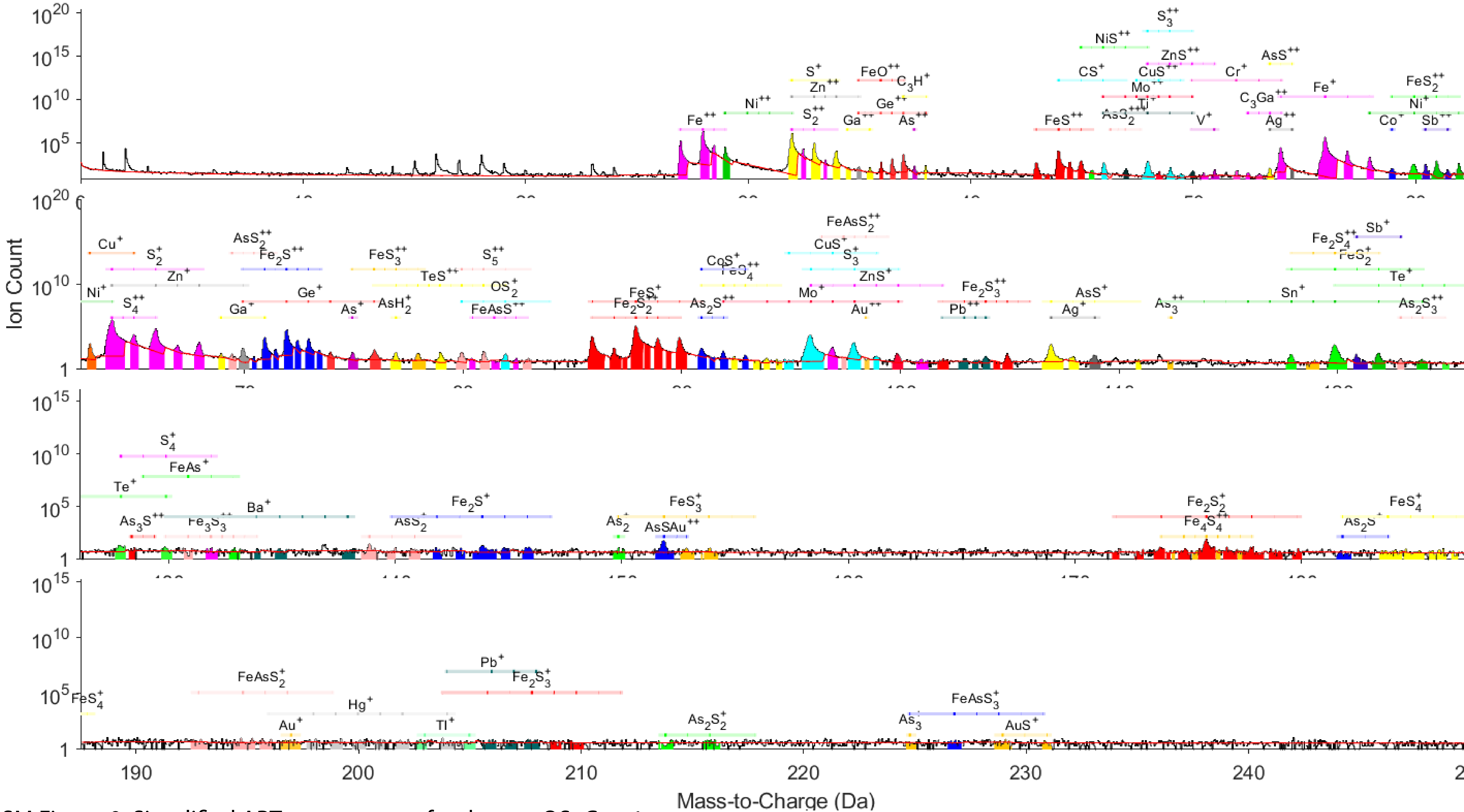

ESM Figure 9: Simplified APT mass spectra for dataset OS_Core1.

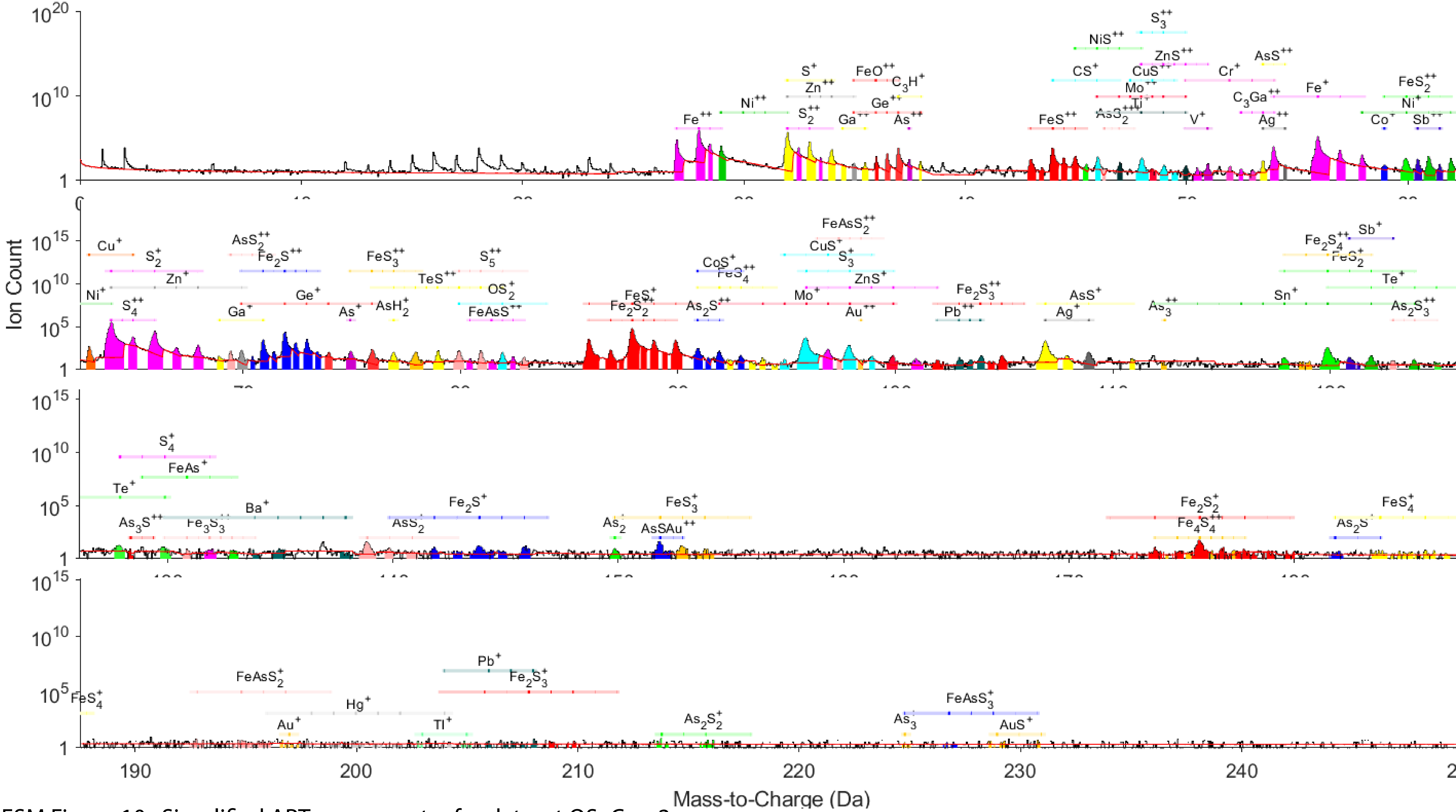

ESM Figure 10: Simplified APT mass spectra for dataset OS_Core2.

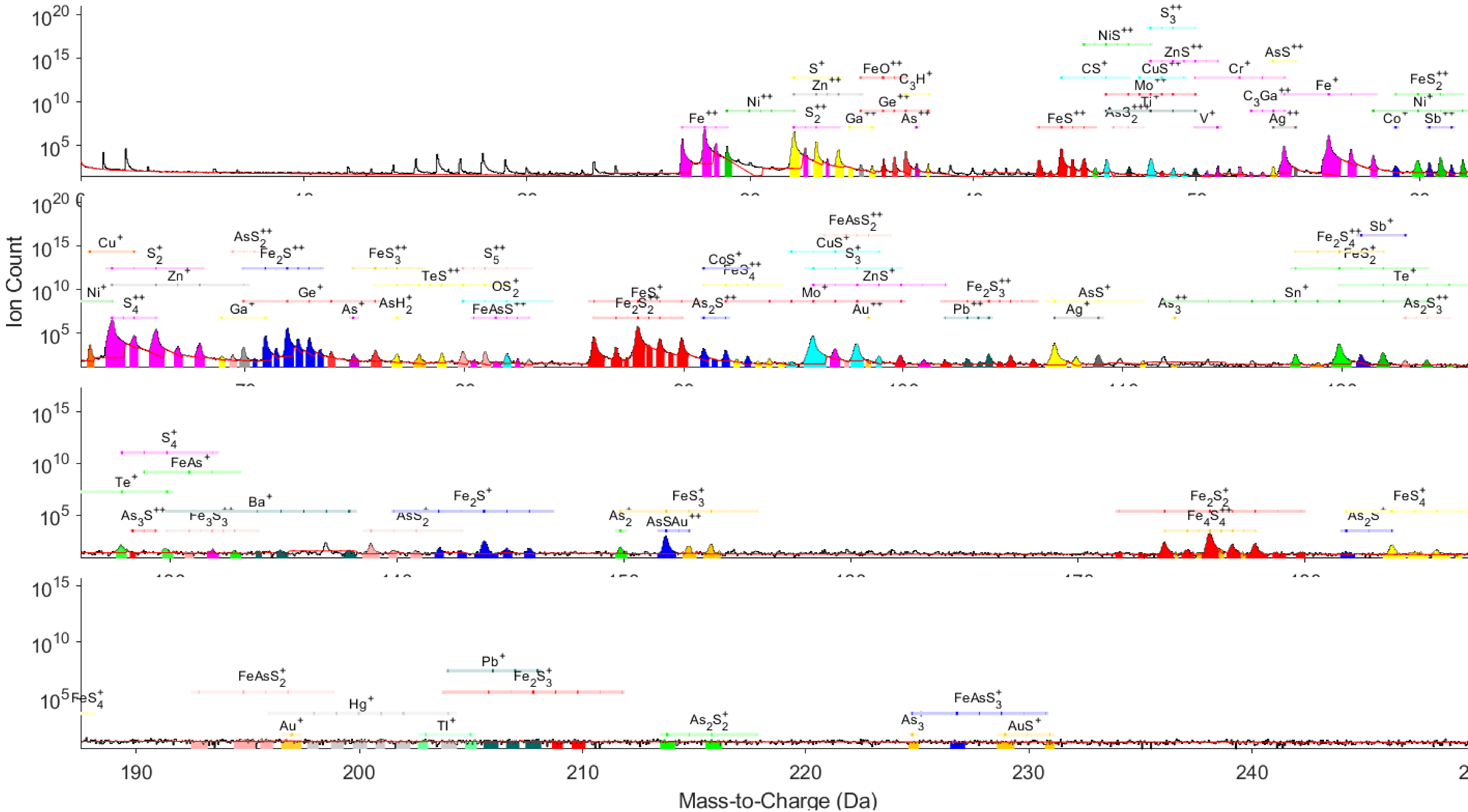

ESM Figure 11: Simplified APT mass spectra for dataset OS_Core3.

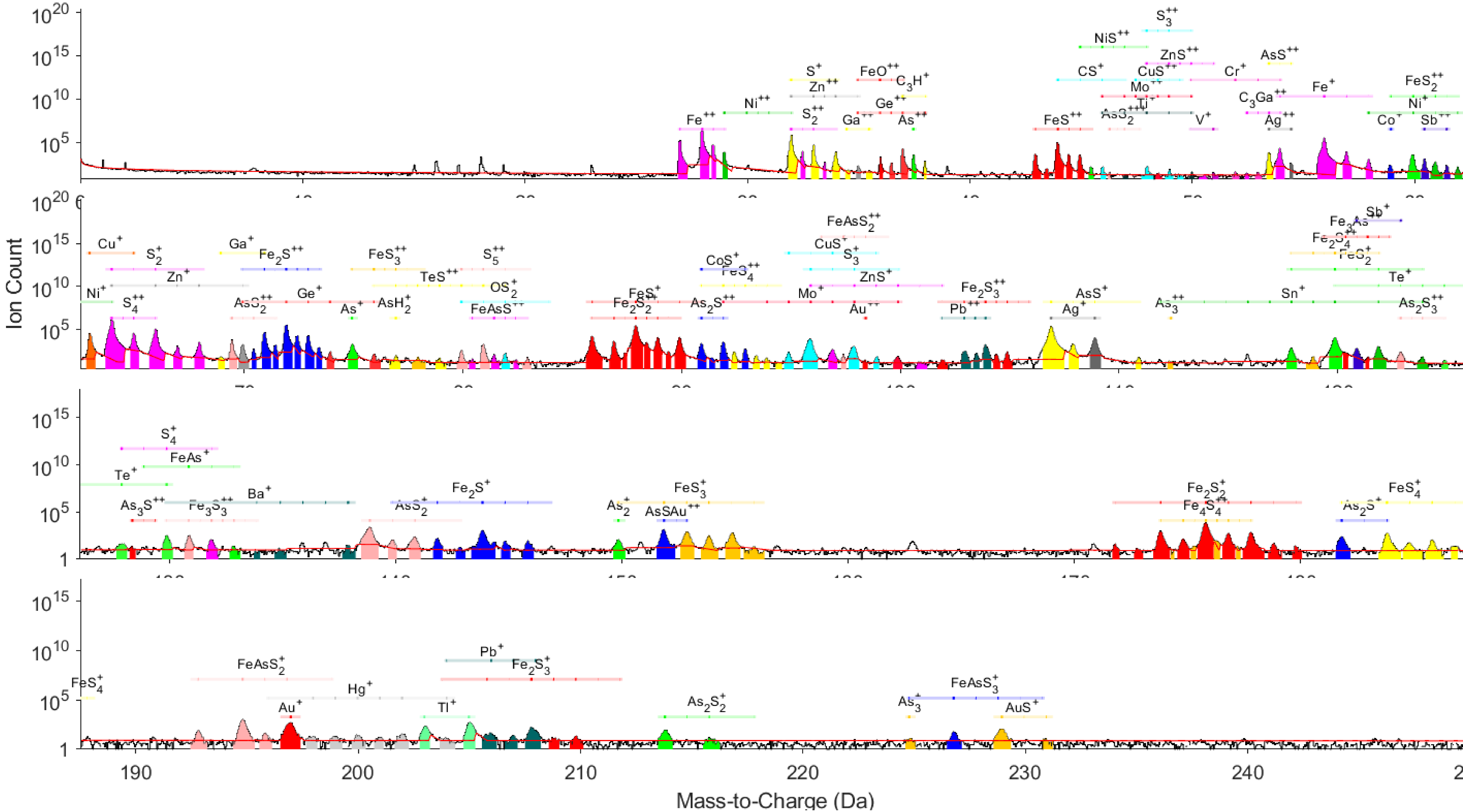

ESM Figure 12: Simplified APT mass spectra for dataset OS_Rim1.

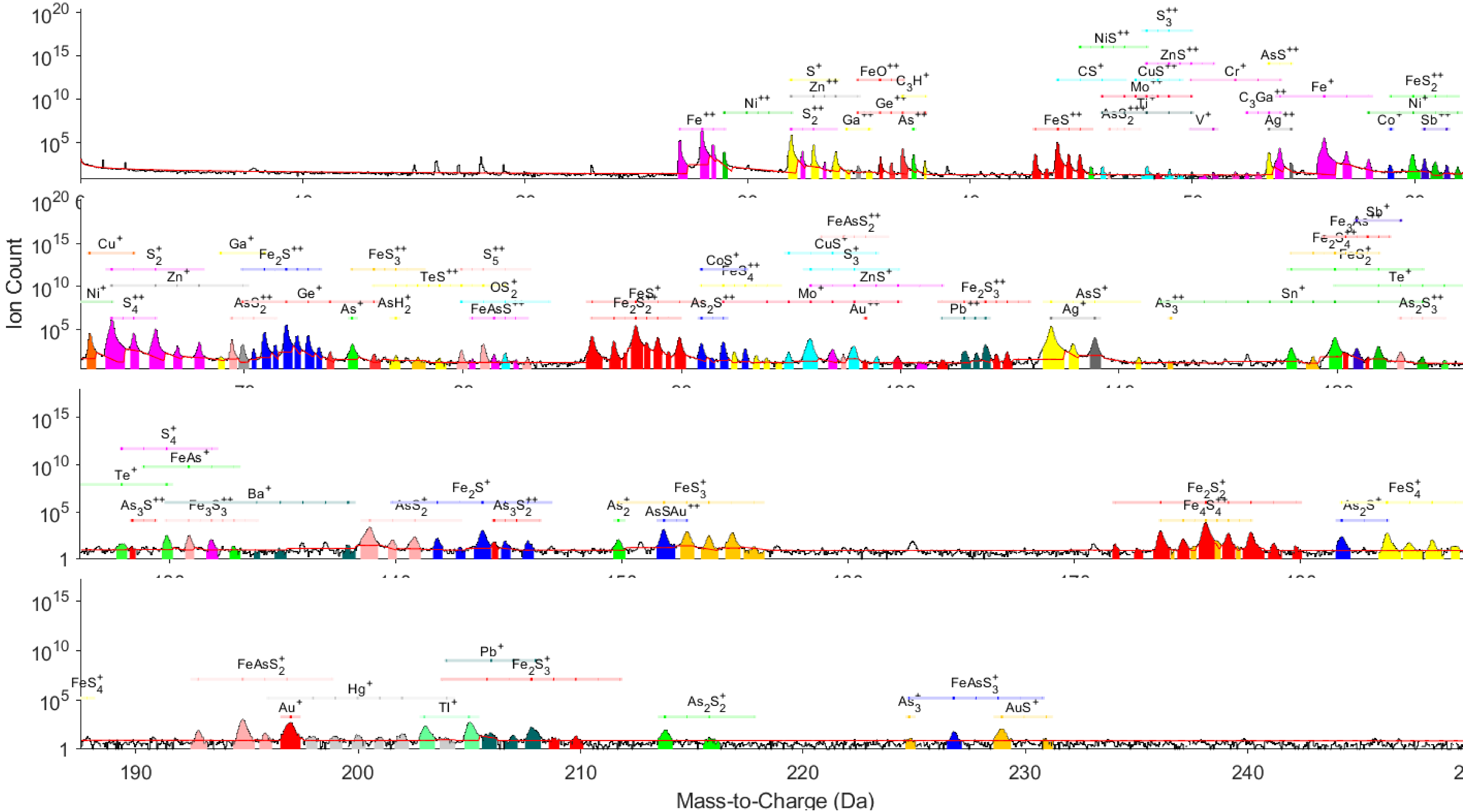

ESM Figure 13: Simplified APT mass spectra for dataset OS_Rim2.

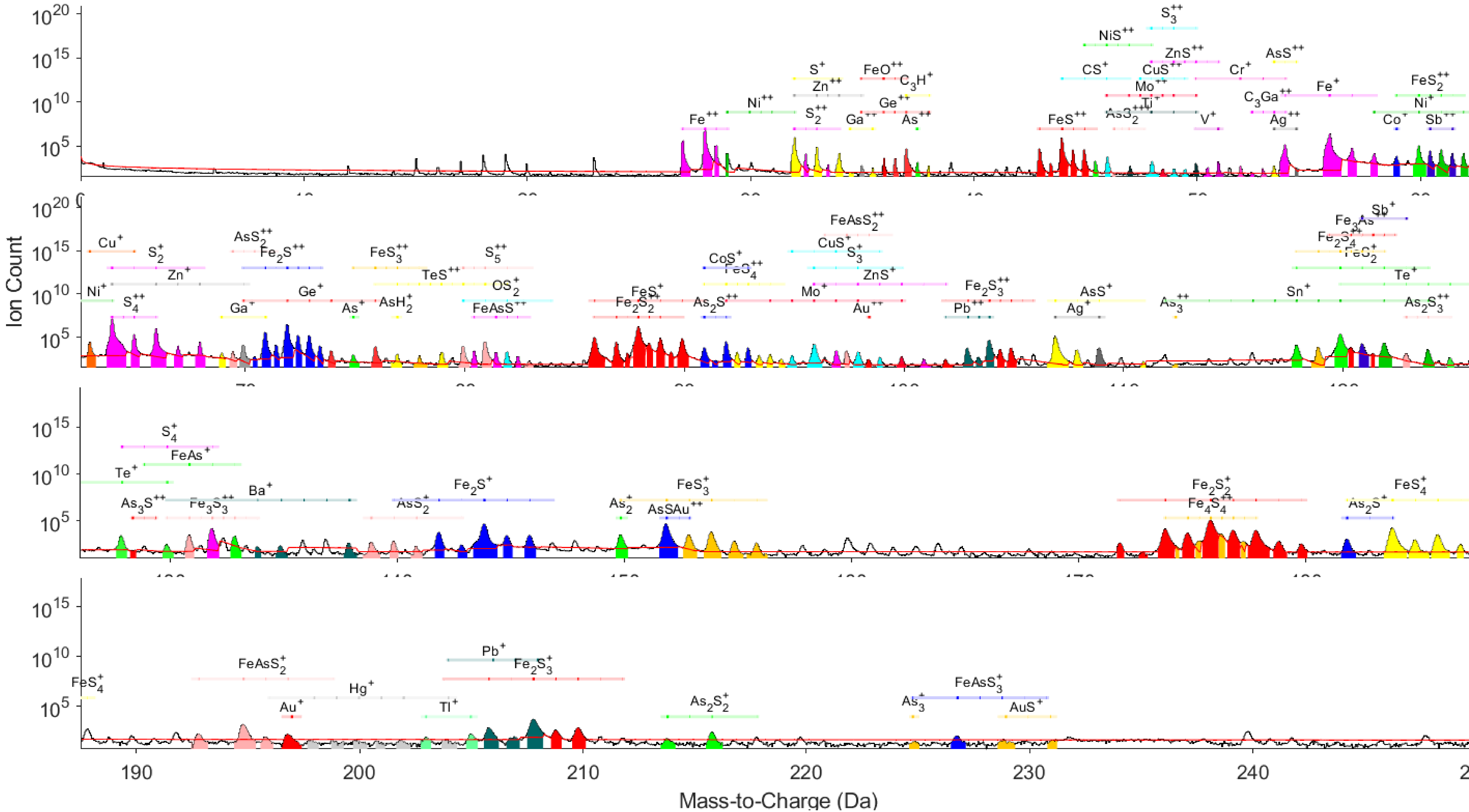

ESM Figure 14: Simplified APT mass spectra for dataset OS_Rim3.
